# Biofabricated Palladium Nanoparticle-Decorated Reduced Graphene Oxide Nanocomposite Using the *Punica granatum* (Pomegranate) Peel Extract: Investigation of Potent In Vivo Hepatoprotective Activity against Acetaminophen-Induced Liver Injury in Wistar Albino Rats

**DOI:** 10.1021/acsomega.3c02643

**Published:** 2023-06-29

**Authors:** Nalinee Kanth Kadiyala, Badal Kumar Mandal, L. Vinod Kumar Reddy, Crispin H. W. Barnes, Luis De Los Santos Valladares, Sireesh Babu Maddinedi, Dwaipayan Sen

**Affiliations:** † Trace Elements Speciation Research Laboratory, Department of Chemistry, School of Advanced Sciences, Vellore Institute of Technology (VIT), Vellore 632014, India; ‡ Cellular and Molecular Therapeutics Laboratory, Centre for Biomaterials, Cellular and Molecular Theranostics, Vellore Institute of Technology (VIT), Vellore 632014, India; § Cavendish Laboratory, Department of Physics, University of Cambridge, Cambridge CB3 0HE, United Kingdom; ∥ Laboratorio de Cerámicos y Nanomateriales, Facultad de Ciencias Físicas, Universidad Nacional Mayor de San Marcos, Ap. Postal 14-0149 Lima, Peru

## Abstract

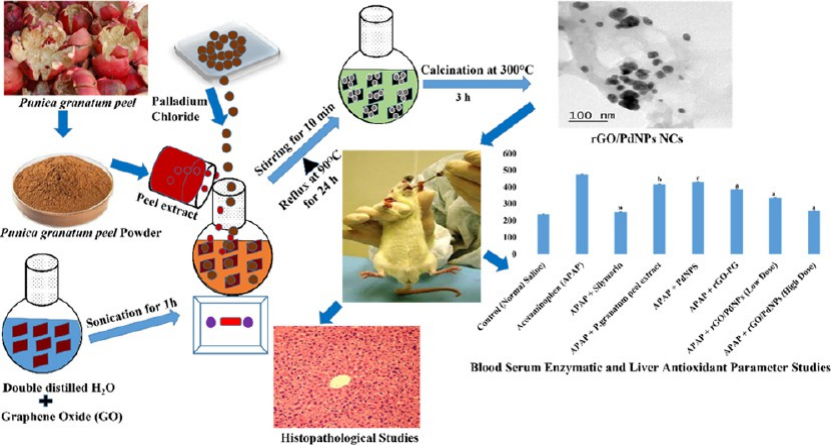

Acute acetaminophen (APAP) toxicity is a predominant clinical problem, which causes serious liver injury in both humans and experimental animals. This study presents the histological and biochemical factor and antioxidant enzyme level changes induced by an acute acetaminophen overdose in Wistar albino rat livers to elucidate the effective hepatoprotective potential of biofabricated palladium nanoparticle-decorated reduced graphene oxide nanocomposites (rGO/PdNPs-NC) compared to silymarin. After detailed characterization of the hepatoprotective potential of the synthesized rGO/PdNPs-NC, the rats were divided into eight groups (*n* = 6): control group (normal saline, 1 mL/kg b.w.), silymarin, *Punica granatum* (pomegranate) peel extract, PdNPs, reduced graphene oxide (rGO-PG), and reduced graphene oxide palladium nanocomposites (rGO/PdNPs-NC, low and high doses) for 7 successive days. The acetaminophen (APAP)-treated group was administered a single dose of acetaminophen (2 g/kg b.w.) on the 8th day. The histopathological results showed that the acetaminophen overdose group exhibited massive intrahepatic hemorrhagic necrosis around the centrilobular region with hepatocytes with vacuolization and swollen cytoplasm found in the liver architecture. This hepatopotential was further assessed by various biochemical parameters such as SGOT, SGPT, ALB, ALP, LDH, direct bilirubin, total bilirubin, and total protein. Also, the antioxidant parameters such as SOD, CAT, MDA, GSH, GRD, and GST were assayed. Rats of groups 7 and 8 showed a significant decrease in SGOT, SGPT, ALP, LDH, direct bilirubin, and total bilirubin (*p* < 0.001), while a significant increase in the final total protein and ALB as compared to group 2 rats (*p* < 0.001) was observed. The antioxidant parameters exhibited that rats of groups 7 and 8 showed a significant (*p* < 0.001) increase in the level of SOD, CAT, GSH, GRD, and GST without affecting the MDA as compared to group 2 rats. Also, the hepatoprotective potential of rGO/PdNPs-NC (low and high doses) was comparable to that of the standard reference drug silymarin. The present study reveals that the rGO/PdNPs-NC possesses significant hepatoprotective activity and acts as an effective and promising curative agent against acetaminophen-induced hepatotoxicity.

## 1 Introduction

The liver, one of the pivotal key organs of the body, is primarily responsible for the metabolism of biomolecular components, supply of coagulation factors, and regulation of the hormonal changes, and it also helps in elimination of detoxicant materials from the body. During the detoxification process, if overwhelmed, the liver cells are damaged and often experience many disorders.^1−3^ Also, it was observed that they are eliminated through the liver, biliary system, and urine, which prevents excessive accumulation in body tissues and organs.^4^ Nowadays, liver diseases are regarded as one of the most serious health ailments, and they can lead to morbidity and mortality all over the world.^5−7^ There are very few conventional drugs, which can stimulate liver function and offer hepatic protection or help in the rejuvenation of hepatic cells.^8−10^

Acetaminophen is one of the important FDA-approved drugs commonly utilized worldwide for its antipyretic or analgesic properties, and its maximal recommended therapeutic dose is 1000 mg for every 4–6 h per individual, i.e., not more than 4 g per day for a consecutive intake (max. 4 mg/day/individual) of 10 days, which is generally considered to be well-tolerated and nontoxic dosage concentrations.^11^ Acetaminophen is rapidly absorbed from the gastrointestinal tract and small intestine and after absorption finally enters the liver through the portal vein. The liver can metabolize acetaminophen to low/nontoxic agents, and at therapeutic levels, 95% of acetaminophen is principally metabolized in the liver by phase II conjugation reactions via glucuronidation and sulfation of its phenolic group.^12−17^ The highly reactive toxic metabolite *N*-acetyl-*p*-benzoquinoneimine (NAPQI) has an extremely short half-life and is rapidly deactivated by irreversible conjugation with the intracellular natural antioxidant glutathione (GSH), forming the 3-glutathionyl conjugate of acetaminophen followed by mercapturic acid, which is excreted in urine.^18^ However, an overdose of acetaminophen would result in saturation of both the sulfation and glucuronidation pathways. Excessive production of the NAPQI metabolite through the CYP2E1 pathway completely depletes the hepatocellular GSH pool^19^ as well as the antioxidant defense system and the hepatocellular regeneration/repair capacity. As a result, the liver becomes more prone toward oxidative stress. Almost 50% of the acetaminophen (APAP)-associated overdoses were accidental poisoning, whereas the other 50% were counted to be intentional (suicide attempts).^12−14^ An obvious sign of hepatotoxicity can be seen by leakage of cellular enzymes into the plasma,^20^ leading to upregulation of the enzyme levels in the serum.

Currently, management of acetaminophen-induced hepatotoxicity is still a challenge to modern pharmacology and medicine due to inadequate treatment of liver damages and serious side effects. Nevertheless, graphene, with a perfect two-dimensional (2D) nanosheet structure embedded with nanoparticles, has been reported as a promising material, which offers several biomedical applications.^21−28^

There are various methods for the synthesis of embedded metal nanoparticles on graphene, graphene oxide (GO), and reduced graphene oxide (rGO) through chemical reduction.^29,30^ However, many of these methods are highly expensive and require huge amounts of energy and hazardous reagents. Therefore, it is important to design bioinspired and ecofriendly greener methods for the synthesis of composites based on metal nanoparticles embedded on GO and rGO and test them as therapeutic agents. Biosynthesis of metal nanoparticles using aqueous biological extracts and environmentally benign methods is mostly practiced in nanotechnology and nanoscience.^31−36^ Greener synthesis methods enjoy the advantages of simple methodology, high yields, environmental friendliness, cost effectiveness, easy work up, and elimination of hazardous reagents.

Among different plant material sources, the *Punica granatum* peel extract has attracted the attention of many researchers for the biosynthesis of silver,^37−42^ gold,^43^ and copper oxide (Cu_2_O) NPs.^44^ The major advantage of the pomegranate peel extract is its abundant source of polyphenol compounds and antioxidants. These components act as better reducing, stabilizing, and capping agents in synthesizing graphene-based nanocomposites. Recently, biofabrication of the palladium nanoparticle-decorated reduced graphene oxide nanocomposite (rGO/PdNPs-NC) has also been reported.^37−39^ The objective of this study was synthesizing Pd NP-decorated reduced graphene oxide (rGO/PdNPs-NC) via one-pot solvothermal reduction of GO and Pd^2+^ ions by using *Punica granatum* peel extract as a green reducing and stabilizing agent. Herein, for the first time, we also report the evaluation of hepatoprotective potential of the synthesized nanocomposite against acetaminophen-induced hepatotoxicity in Wistar albino rats.

## 2 Materials and Methods

### 2.1 Chemicals and Reagents

The chemicals and reagents used in this work were graphite powder (100 mesh, 99.9%), potassium permanganate (KMnO_4_, >99%), sodium nitrate (NaNO_3_, AR grade, Qualigens, India), hydrogen peroxide (H_2_O_2_, 30%), sulfuric acid (H_2_SO_4_, 98%), hydrogen chloride (HCl, 36%), palladium, paracetamol (MICRO LABS Ltd., Mamring, South Sikkim, India), silymarin (MICRO LABS Ltd., Mamring, South Sikkim, India), diagnostic kits for serum glutamate pyruvate transaminase (SGPT), serum glutamate oxaloacetate transaminase (SGOT), alkaline phosphatase (ALP) (BioSystems S.A. Costa Brava, Barcelona, Spain), total protein (TP), albumin, bilirubin (Coral Clinical Systems, Dr. Antonio Do Rego Bagh, Goa, India), lactate dehydrogenase (LDH) (ANAMOL LABORATORIES Ltd., Palghar, India), malonaldehyde (MDA), glutathione (GSH), catalase (CAT), superoxide dismutase (SOD), glutathione reductase (GRD), and glutathione-*s*-transferase (GST). All other organic chemicals or reagents are from Sigma-Aldrich, Bangalore, India. Double-distilled water was used throughout the experiment.

### 2.2 Preparation of the *Punica granatum* (Pomegranate) Peel Extract

Fresh and matured pomegranate fruits were procured from a local fruit market in Vellore, Tamil Nadu, India. First, the fruits were thoroughly washed with running tap water followed by double-distilled water to remove adhering dust particles. Then, the pomegranate peels were well separated from the arils/seeds of the pomegranate fruit and allowed to air dry in shade at 25–30 °C for about one week. The dried peels were further finely powdered by using a laboratory mechanical blender. 50 g of dried powdered peels was soaked in 500 mL of double-distilled water in an Erlenmeyer flask and refluxed for 1 h at 90 °C in a hot-water bath and allowed to cool to room temperature and filtered using Whatmann filter paper No. 4. The filtered aqueous peel extract solution was refrigerated at 4 °C until use.

### 2.3 Green Synthesis of rGO/PdNPs-NC, rGO-PG, and PdNPs

Graphene oxide (GO) used for the preparation of rGO/PdNPs-NC was synthesized from natural graphite powder by a modified Hummers method.^45^ GO (1.0 mg/mL) homogeneous dispersion solution in a 500 mL round-bottom flask was prepared by 1 h of sonication. Then, 100 mL of 1 mM aqueous PdCl_2_ solution was added dropwise into the mixture under vigorous stirring. Subsequently, the flask was mounted with a cooling condenser and magnetic stir bar, which is heated at 90 °C. The rGO/PdNPs-NC was prepared by a green synthesis method using the aqueous *Punica granatum* (pomegranate) peel extract, which is rich in phytochemicals such as anthocyanins, free ellagic acid, ellagic acid glycosides, ellagitannins, gallotannins, and punicalagin. These phytochemicals may act as biologically effective reducing and stabilizing agents.^39−41^

**Scheme 1:**
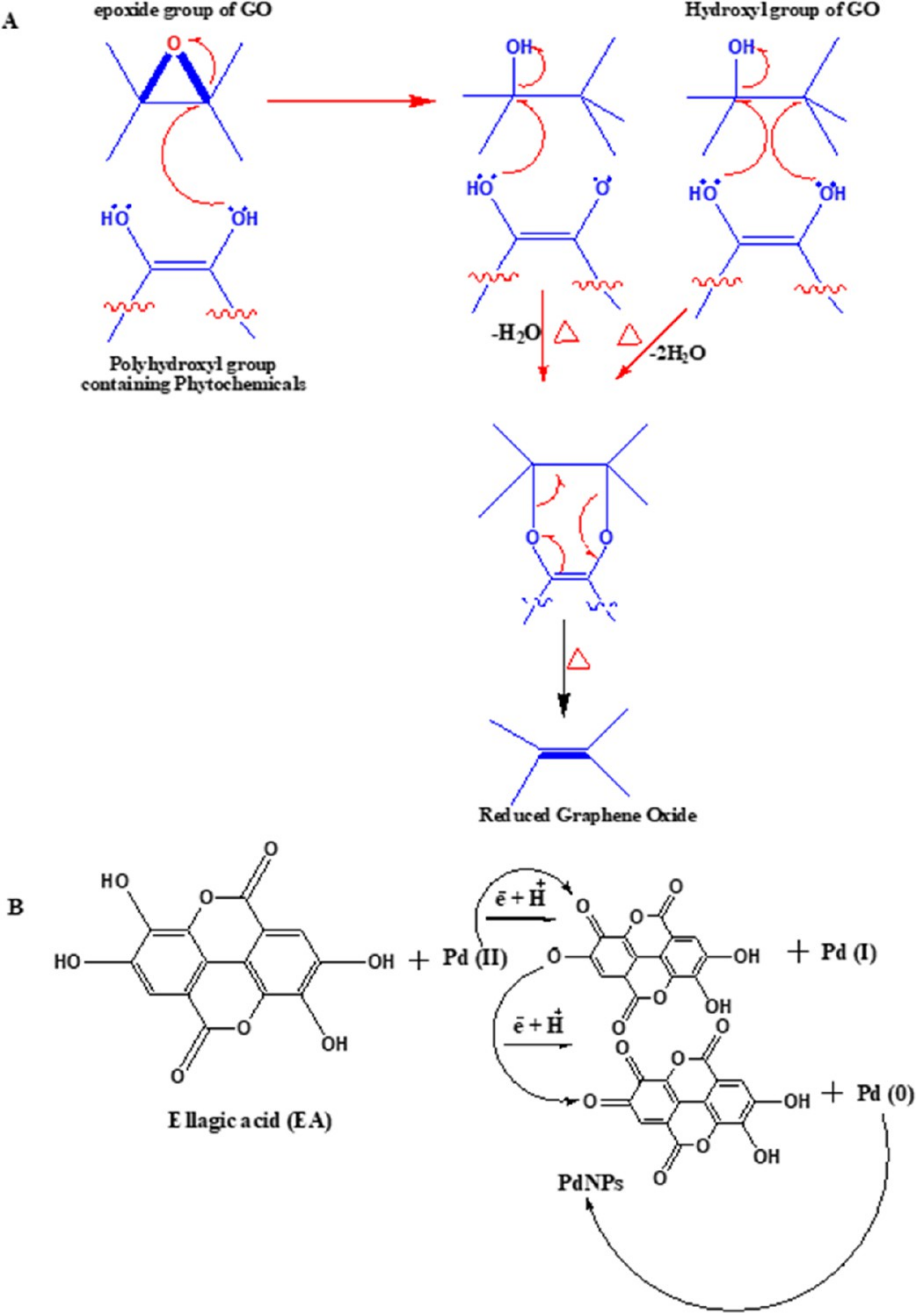
Predicted Reaction Mechanism of the Green Reduction of (A) GO to rGO-PG and (B) Pd Ions to PdNPs and Uniform Decoration of PdNPs onto the Surface of rGO/PdNPs-NC

The basic mechanism for the synthesis of rGO/PdNPs-NC is illustrated in Scheme 1. The GO possesses majorly three types of oxygen functionalities such as hydroxyl, epoxy, and carboxylic groups.^46−48^ Epoxy groups undergo a ring opening reaction with the loss of water molecules, whereas hydroxyl functionalities facilitate condensation for ring formation followed by ring cleavage. Moreover, the polyphenols that are present in the *Punica granatum* (pomegranate) peel extract are primarily responsible for the simultaneous reduction of Pd^2+^ ions and GO and also for the growth and stabilization of PdNPs, which result in uniform decoration of the synthesized PdNPs onto the surface of rGO, forming rGO/PdNPs-NC (Scheme 1).

For the synthesis of the rGO/PdNPs-NC, 50 mL of the above-mentioned aqueous solution was added to the suspension following further stirring for 24 h at 90 °C. Afterward, the resultant black powder of rGO/PdNPs-NC was collected by centrifugation and repeatedly washed with deionized water in order to remove the excess aqueous peel extract residue and suspended into water for sonication. The suspension was centrifuged at 10 000 rpm for 30 min. The final product was collected and dried in a hot-air oven at 80 °C overnight. The dried black powder was calcined at 300 °C for 3 h to obtain the final rGO/PdNPs-NC. The PdNPs were synthesized from the palladium chloride precursor under similar experimental conditions without graphene oxide (GO). The rGO-PG can be obtained further by the effective reduction of graphene oxide (GO) using the aqueous peel extract following strictly similar experimental conditions for the preparation of rGO/PdNPs-NC.

### 2.4 Material Characterization

The crystallinity studies of the synthesized nanocomposites were evaluated by X-ray diffraction (XRD) analysis on a Bruker D8 Advance diffractometer with Cu Kα (λ = 1.54 Å) radiation at 40 kV and 20 mA. The functional groups of the synthesized nanocomposite were analyzed using a Fourier transform infrared spectrophotometer in attenuated total reflectance (JASCO ATR-FTIR 4100). A Carl Zeiss scanning electron microscope (SEM) was used to investigate the morphology and particle dispersion of the produced nanocomposite. The chemical composition of the prepared nanocomposite was measured by energy dispersive X-ray spectroscopy (EDS) performed using SEM. Transmission electron microscopy (TEM) images and selected-area electron diffraction (SAED) patterns were examined using an HR-TEM (JEOL JEM 2100). X-ray photoelectron spectra were measured using monochromatized Al Kα at 1486.6 eV with a nominal spot size of 400 μm (ESCA-3000 VG Scientific U.K.). Peak fitting was performed using XPS Peak 4.1 software.

### 2.5 Experimental Animals

The experimental study was performed on healthy, sexually mature male albino rats (Wistar strain). They weighed 220–250 g and 8–10 weeks old, and they were kept in the VIT animal house, Vellore, Tamil Nadu, India. The animals were acclimatized for a minimum of 7 days prior to use and maintained in standard laboratory conditions. The rats were housed separately in clean and dry polypropylene cages filled with sterile paddy husk as bedding throughout the experiment at a constant ambient temperature (25 ± 2 °C) and relative humidity (40–60%) with a 12 h light/dark cycle and allowed free access to standard commercial pellet diet and purified drinking water throughout the housing period of the experiment. Animals were subjected to fasting overnight by withdrawing the food 18–24 h before the experiment, although free access to water *ad libitum* was allowed. All animal care and experimental protocols were performed in accordance with the guidelines set forth by the “Committee for the Purpose of Control and Supervision” (CPCSEA), and ethical clearance was approved by the Institutional Animal Ethical Committee (IAEC) of the Vellore Institute of Technology (VIT), Vellore, Tamil Nadu, India (Approval NO.: VIT/IAEC/12/July23/37).

### 2.6 Evaluation of Hepatoprotective Activity

#### 2.6.1 Induction of Acetaminophen-Induced Hepatotoxicity

The animals were randomly divided into eight groups and housed in polypropylene cages (*n* = 6/group) as follows:

Group 1: the normal control (NC) group received physiological saline (1 mL/kg b.w.) at a single daily dose orally via gavage for 7 consecutive days and served as the healthy control.

Group 2: the hepatotoxic control (APAP) group received physiological saline (1 mL/kg b.w.) at a single daily dose orally via gavage for 7 consecutive days, and then, hepatotoxicity was induced by oral administration of acetaminophen (2 g/kg b.w.) in a single dose on day 8.

Group 3: the positive/standard control group received the silymarin standard drug (100 mg/kg b.w.) at a single daily dose orally via gavage for 7 consecutive days, and then, hepatotoxicity was induced by oral administration of acetaminophen (2 g/kg b.w.) in a single dose on day 8.

Group 4: the test group received the *Punica granatum* (pomegranate) peel extract (100 mg/kg b.w.) at a single daily dose orally via gavage for 7 consecutive days, and then, hepatotoxicity was induced by oral administration of acetaminophen (2 g/kg b.w.) in a single dose on day 8.

Group 5: the test group received PdNPs (100 mg/kg b.w.) at a single daily dose orally via gavage for 7 consecutive days, and then, hepatotoxicity was induced by oral administration of acetaminophen (2 g/kg b.w.) in a single dose on day 8.

Group 6: the test group received rGO-PG (50 mg/kg b.w.) at a single daily dose orally via gavage for 7 consecutive days, and then, hepatotoxicity was induced by oral administration of acetaminophen (2 g/kg b.w.) in a single dose on day 8.

Group 7: the test group received rGO/PdNPs-NC (25 mg/kg b.w.) (low dose) at a single daily dose orally via gavage for 7 consecutive days, and then, hepatotoxicity was induced by oral administration of acetaminophen (2 g/kg b.w.) in a single dose on day 8.

Group 8: the test group received rGO/PdNPs-NC (50 mg/kg b.w.) (high dose) at a single daily dose orally via gavage for 7 consecutive days, and then, hepatotoxicity was induced by oral administration of acetaminophen (2 g/kg b.w.) in a single dose on day 8.

On the 9th day of the experiment, i.e., 24 h after the last dosing by acetaminophen, the overnight-fasted rats from each of the groups were anesthetized using chloroform 3 min prior to sampling. Blood samples were collected into dry sterilized plain centrifuge tubes without any anticoagulants by cardiac puncture, allowed to clot for 1 h at room temperature, and centrifuged at 3000 rpm at 4 °C for 15 min to obtain sera. The serum samples were isolated and stored at −20 °C for further assessment of various biochemical or enzyme activities. Animals were then euthanized by cervical dislocation, and the livers were dissected out and washed immediately at least twice with ice-cold isotonic saline solution (0.9% sodium chloride) to remove as much blood as possible, blotted, and dried. A segment from the midpoint lobe of the liver was fixed in 10% formalin solution for histopathological investigation. The second part from the same lobe, 10% (w/v) tissue, was homogenized in 0.1 M phosphate buffer saline (PBS) at (pH = 7.4). The homogenates were centrifuged at 10 000 rpm at 4 °C for 30 min, and the resultant post-mitochondrial supernatant (PMS) was separated and stored at −70 °C until usage for determination of antioxidant enzyme activity.

#### 2.6.2 Assessment of Hepatoprotective Biochemical Parameters

Liver hepatotoxicity was assessed by estimating the enzymatic activities of serum aspartate aminotransferase (AST), alanine aminotransferase (ALT), alkaline phosphatase (ALP), lactate dehydrogenase (LDH), albumin (ALB), total protein (TP), total bilirubin (TB), and direct bilirubin (DB) levels, carried out using a UV spectrometer and commercially available enzymatic biochemical diagnostic kits following the manufacturer’s instructions.

#### 2.6.3 Assessment of Antioxidant Biochemical Parameters

The post-mitochondrial supernatant (PMS) was used for the measurements of MDA, glutathione (GSH), catalase (CAT), superoxide dismutase (SOD), glutathione reductase (GRD), and glutathione-*s*-transferase (GST). All these enzymes’ activity was determined with respect to the manufacturer’s instructions provided within the commercial kits.

### 2.7 Histopathological Examination

For histopathological examination studies, the slices of the liver from each group were stored in 10% buffered neutral formalin at (pH = 7.4). They were then dehydrated through an ascending grade series of alcohol (70–100%) followed by clearing in xylene and embedded in paraffin at 58 °C for 4–5 h. Thin sections of 5 μm thickness of the liver were cut using a rotary microtome. The sections were stained using hematoxylin–eosin dye and examined under a high-resolution microscope (Olympus, Japan) to observe the histopathological changes in the liver including fatty infiltration, cell necrosis, lymphocyte infiltration, and ballooning generation. All samples were examined, and photomicrographs were taken.

### 2.8 Statistical Analysis

All experiments were done in triplicate, and the results were expressed as mean ± standard error. Statistical analysis was conducted using one-way analysis of variance (ANOVA) with Tukey–Kramer multiple comparisons. Difference was considered significant at ^a^*p* < 0.001 and ^b^*p* < 0.01 when compared to the control group vs paracetamol-treated group and test group vs paracetamol-treated group.

## 3 Results

### 3.1 Characterization of the rGO/Pd-NC

#### 3.1.1 X-ray Diffraction (XRD) Studies

**Figure 1:**
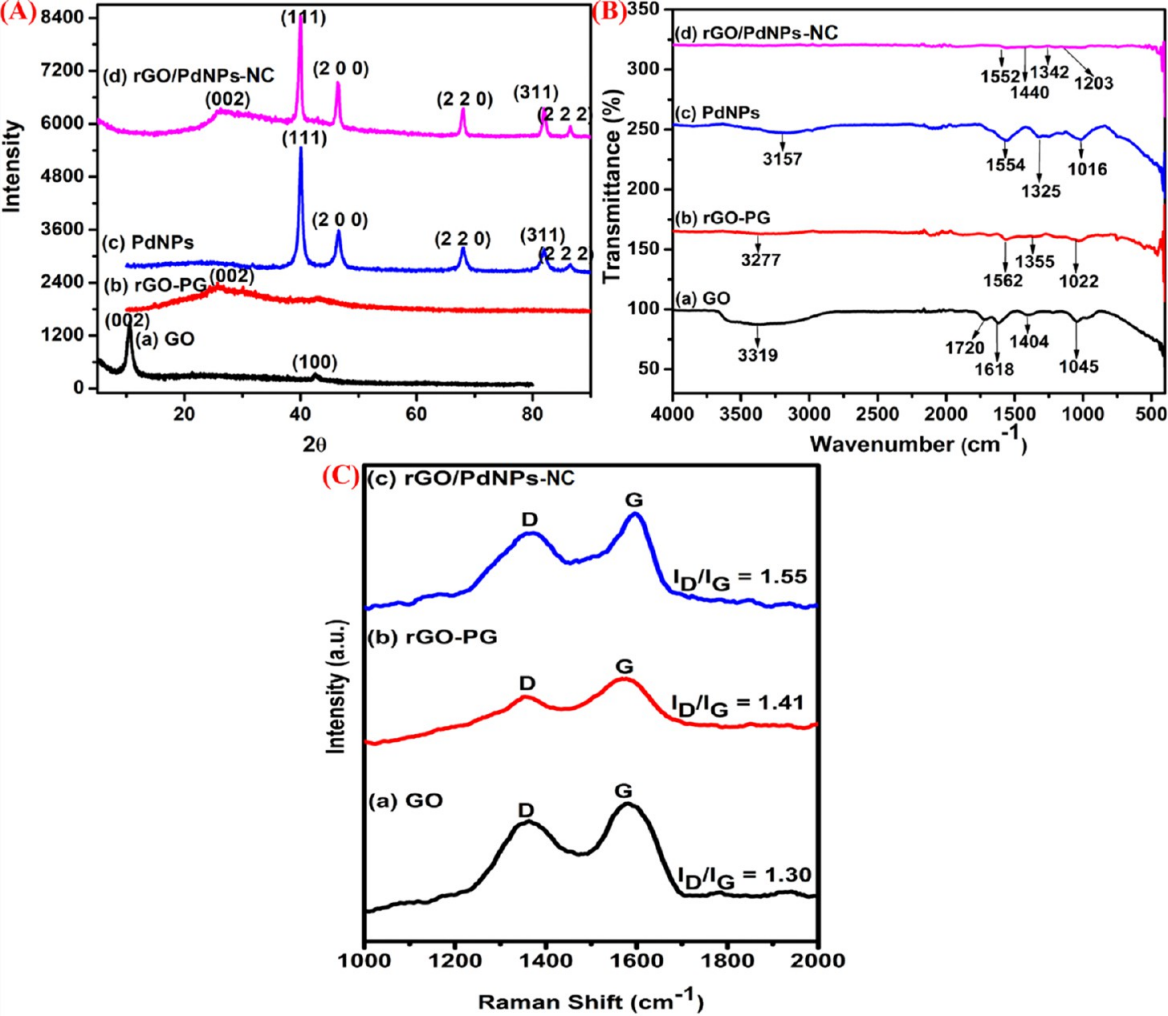
XRD patterns of graphene oxide (GO) 1A(a), reduced graphene oxide (rGO-PG) 1A(b), PdNPs 1A(c), and reduced graphene oxide palladium nanoparticle nanocomposite (rGO/PdNPs-NC) 1A(d); FTIR spectra of graphene oxide (GO) 1B(a), reduced graphene oxide (rGO-PG) 1B(b), PdNPs 1B(c), and reduced graphene oxide palladium nanoparticle nanocomposite (rGO/PdNPs-NC) 1B(d); and Raman spectra of graphene oxide (GO) 1C(a), reduced graphene oxide (rGO-PG) 1C(b), and reduced graphene oxide palladium nanoparticle nanocomposite (rGO/PdNPs-NC) 1C(c).

The XRD diffractograms of the prepared GO, rGO-PG, PdNPs, and rGO/PdNPs-NC measurements are shown in Figure 1. Figure 1A(a) shows the XRD patterns of the as-produced graphene oxide (GO), in which the intense diffraction peak observed at 2θ = 10.4° is characteristic for the (002) reflection plane of GO. This characteristic peak suggests the existence of intercalated water molecules and different kinds of oxygen species and functional groups in the AB-stacked graphitic layers. A small peak seen at 2θ = 42.5°, (100) reflection plane, corresponds to the small amount of hexagonal-structured graphite phases, which are still present on the surface of GO. It is noteworthy that the greener chemical reduction process initiated by the *Punica granatum* (Pomegranate) peel extract caused this peak to decrease, leading to a broad peak shift from 2θ = 10.4° to 2θ = 25.7°, which exactly matches with the (002) reflection plane of rGO-PG. This shift also suggests the successful removal of carboxyl, hydroxyl, and some other oxygen-containing functional groups from the rGO surface when subjected to reduction in the presence of plant extract materials (Figure 1A(b)).^49,50^

Note the crystalline nature of the pure PdNPs and PdNPs decorated on the reduced graphene oxide (rGO) nanocomposite (see Figure 1A(c) and (d)) revealing major diffraction peaks at 2θ = 40.0, 46.4, 67.9, 81.8, and 86.5°, which can be indexed to the (111), (200), (220), (311), and (222) crystallographic planes of the face-centered cubic (fcc) crystalline structure of the metallic palladium (Pd^0^) nanoparticles (JCPDS No: 89–4897), respectively. The characteristic, broad, and small peak at 2θ = 26.1° can also be noticed, corresponding to the (002) reflection plane of reduced graphene oxide (rGO) in the XRD pattern of the nanocomposite. It indicates the successful conversion of graphene oxide (GO) to reduced graphene oxide (rGO) with the formation of the (sp^2^ carbon) graphene network during the reduction process. The uniformly distributed metallic PdNPs (Pd^0^) on the reduced graphene oxide (rGO) surface act as spacers and prevent the graphene sheets from undergoing the restacking phenomenon. Hence, XRD studies reveal the successful formation of rGO/PdNPs-NC.

#### 3.1.2 Attenuated Total Reflectance-FTIR (ATR-FTIR) Spectroscopy Studies

The attenuated total reflectance–Fourier transform infrared (ATR-FTIR) spectroscopy technique was used to investigate the functional and chemical structural alterations that occurred between GO, rGO-PG, PdNPs, and rGO/PdNPs-NC in the presence of various biomolecules that exist in the *Punica granatum* (pomegranate) peel extract, which are the prime reason for the effective reduction of both graphene oxide (GO) to reduced graphene oxide (rGO) and palladium ions into palladium metal (Pd^0^) nanoparticles. The GO, rGO-PG, PdNP, and rGO/PdNPs-NC FTIR spectra are presented in Figure 1B. In the spectrum of graphene oxide (GO, Figure 1B(a)) obtained after oxidation of graphite, the characteristic absorption peaks range at 3319, 1720, 1618, 1404, and 1045 cm^–1^, and they are assigned to the stretching mode of −OH groups, C=O stretching vibration mode of the (COOH) group, sp^2^ characteristic stretching of C=C in GO, and vibrational stretching of C–OH and C–O groups, respectively. Following the reduction of GO by the *Punica granatum* (pomegranate) peel extract (see the rGO-PG spectrum in Figure 1B(b)), the characteristic peak intensities related to oxygen-containing functional groups such as O–H stretching at 3319 cm^–1^ shift to lower intensity observed at 3277 cm^–1^; the peaks corresponding to the C=C and C=O stretching vibration mode of carboxylic groups completely disappear, and the peak intensity of the C=C stretching vibration mode at 1562 cm^–1^ slightly increases. These results confirm that the peel extract acts as an effective reducing agent and is able to bring changes in terms of both functional as well as surface transformations of graphene sheets from hydrophilic to hydrophobic nature.^51−53^

Figure 1B(c, d) displays the FTIR spectra of the PdNP and rGO/PdNPs-NC samples. The FTIR spectrum for the rGO/PdNPs-NC (Figure 1B(d)) shows a similar spectrum to that for rGO-PG but without the C=O and C=C stretching vibration mode of carboxylic groups located at 1618 and 1720 cm^–1^ or the additional peaks at 3319 and 1045 cm^–1^ corresponding to the O–H stretching and C–O stretching peaks of GO, respectively, indicating that GO was reduced to rGO during the formation of rGO/PdNPs-NC. In contrast, the FTIR spectrum obtained for the PdNPs (Figure 1B(c)) illustrates the functional groups that are present after the green reduction of palladium ions and the formation of metallic palladium (Pd^0^) nanoparticles. Furthermore, the peaks located at 3277, 1554, 1325, and 1016 cm^–1^ correspond to the O–H functional group, C=C stretching vibration, carboxyl O–H, and epoxy or alkoxy C–O functional groups, respectively.

The polyphenols, flavonoids, alcohols, proteins, aldehydes, carboxylic acids, ketones, amides, and other biomolecules present in the *Punica granatum* (pomegranate) peel extract are responsible for the reduction of the GO and palladium ions. Green reduction takes place by the adsorption of these biomolecules onto the surface of palladium ions due to the absence of strong ligating agents. Thus, the π-electrons of the (C=O) carbonyl group from the “C” rings of flavonoids in a reduction/oxidation system can transfer to the metal ion-free orbital, ultimately leading to the conversion of the metal ion into its free metal state.^54,55^ These reaction mechanism properties have already been well exploited in the preparation of Pd NPs^56^ and Au/Pd NPs.^57^ In the case of GO, this medium contains a bulk quantity of oxygen-containing functional groups such as epoxide, carboxylic acids, and alcohols; these moieties provide excellent aqueous dispersibility and also act as seeded sites for incoming palladium nanoparticles.

Therefore, based on the above-mentioned FTIR data, we could infer that the polyphenols, flavonoids, aldehydes, ketones, amide, proteins, amides, and carboxylic acids belonging to the main phytochemical components of the peel extract of *Punica granatum* (pomegranate) should play an important role in both the reduction of Pd(II) ions and binding capacity toward the reduced graphene oxide (rGO) surface. In this scenario, the *Punica granatum* (pomegranate) peel extract does not only act as a reducing agent but also as a stabilizing agent for the formed rGO/PdNPs-NC.

#### 3.1.3 Raman Spectroscopic Studies

Raman spectroscopy is one of the most powerful and highly sensitive technique that has been employed worldwide for obtaining valuable information regarding structural defects in carbon materials, especially graphene. The estimation of the quality and in-depth electronic properties of graphene includes the information of the defect structure, disorder, doping levels, and defect density.^58,59^Figure 1C shows the comparison of Raman spectra for the GO, rGO-PG, and rGO/PdNPs-NC samples. In the Raman spectrum, graphene exhibits two characteristic features: the D-band at approximately 1350 ± 20 cm^–1^ and the G-band at approximately 1550 ± 20 cm^–1^. Typically, the D-band is attributed to sp^3^-hybridized carbon related to defects and impurities that persist at the edges of the graphene matrix or graphene lattice, whereas the G-band is assigned to the sp^2^-hybridized (C=C) carbon bond stretching in the graphene lattice.^60^ The intensity ratio of D and G bands implies the number of structural defects or disorder that exists in the graphene network and is inversely proportional to the degree of graphitization.^61^ Whenever graphene nanosheets are doped with different metal nanoparticles such as Au, Ag, Pd, and Pt, it results in the shift of peak positions; the D-band intensity increases relatively to that of the G-band, and the G-band shifts to larger wavenumbers.^62,63^

The intensity ratios (*I*_D_/*I*_G_) for the green-synthesized graphene nanocomposite materials GO, rGO-PG, and rGO/PdNPs-NC were calculated and are depicted in Figure 1C. The peak intensity ratio of the “D” and “G” bands for the rGO-PG sample (*I*_D_/*I*_G_ = 1.41, see Figure 1C(b)) is observed to be slightly higher than that obtained for the GO sample (*I*_D_/*I*_G_ = 1.30, see Figure 1C(a)). This can be attributed to the effective removal of oxygen functional species or groups from the GO sheets, which leads to a decrease in size of the in-plane sp^2^ domains. After effective deposition of PdNPs onto the surface of the reduced graphene oxide to the formation of rGO/PdNPs-NC, *I*_D_/*I*_G_ = 1.55 (see Figure 1C(c)). This larger value was expected than that for the initial GO since more defects have been introduced to the rGO/PdNPs-NC, and these could also act as anchoring sites for attracting the Pd metal (Pd^0^) nanoparticles. Hence, it presents a larger *I*_D_/*I*_G_ ratio when compared to those for the rGO-PG and GO nanosheets. Note also that the Raman spectrum for the exfoliated GO exhibits the in-plane vibration of the graphitic lattice at 1580 cm^–1^ (G-mode), whereas the spectrum for the rGO/PdNPs-NC nanocomposite shows a broad and shifted G-band (1588 cm^–1^) and the D-band with high intensity at 1367 cm^–1^. The shift of the G band for the rGO/PdNPs-NC nanocomposite is caused due to the presence of isolated double bonds, which resonate at higher frequency than that for graphite.^64^ The spectrum of the as-produced rGO-PG exhibits a shifted D-band at 1347 cm^–1^ compared to that for GO (1362 cm^–1^), indicating the introduction of defects and disorders into the sample of the in-plane sp^2^ domains.^65^ The above-mentioned observations clearly demonstrate the successful reduction of GO to rGO as well as formation of the rGO/PdNPs-NC nanocomposite.

#### 3.1.4 X-ray Photoelectron Spectroscopy (XPS) Studies

**Figure 2:**
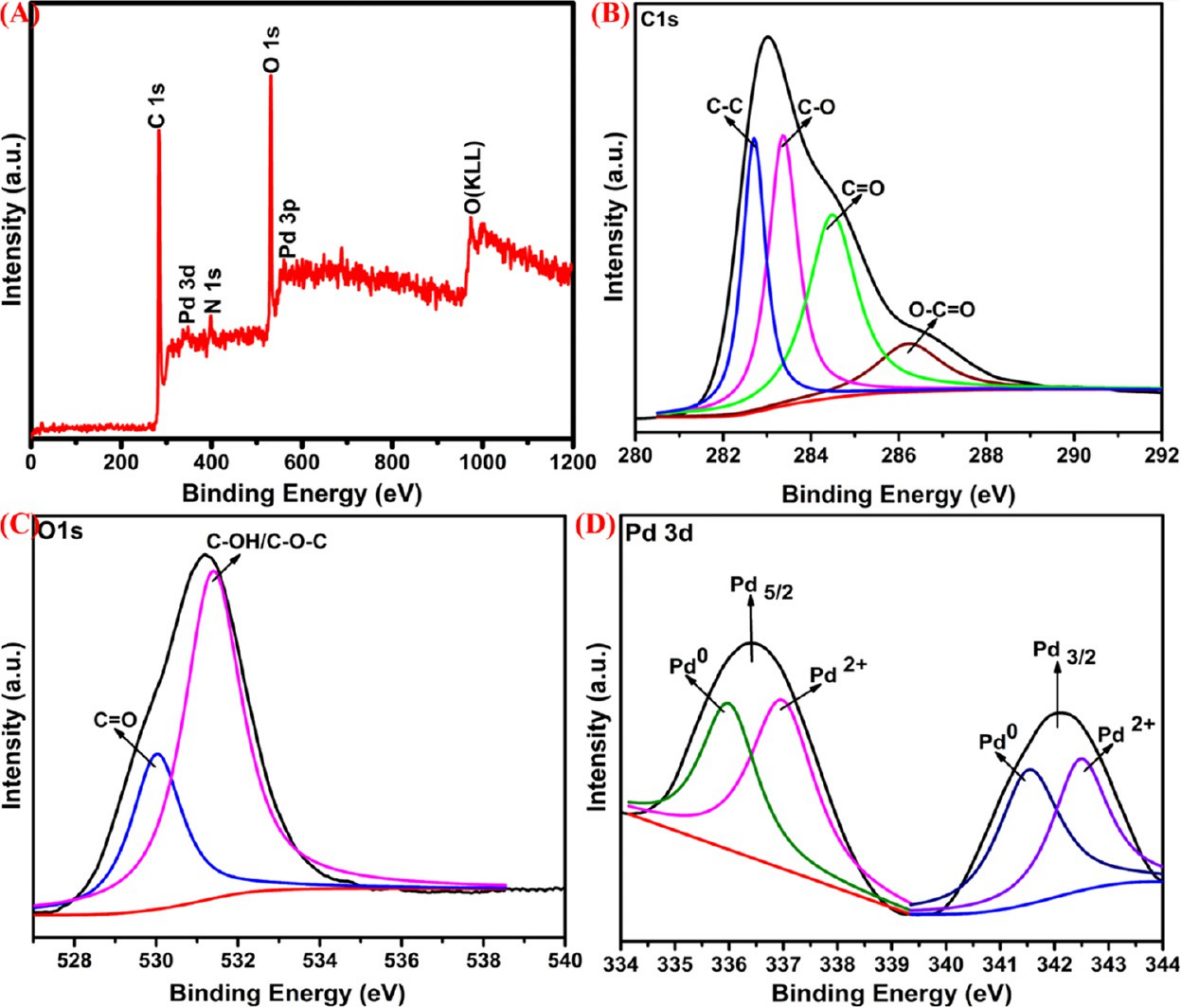
XPS spectra of rGO/PdNPs-NC: overall XPS survey spectrum of rGO/PdNPs-NC (A), high-resolution C 1s scan (B), high-resolution O 1s scan (C), and high-resolution Pd 3d XPS core level spectral region of rGO/PdNPs-NC (D).

X-ray photoelectron spectroscopy (XPS) is a powerful technique employed to explore detailed information regarding the structure, elemental composition, oxidation states, and binding energies of the metal core electrons of surfaces. The XPS spectra of the as-synthesized rGO/PdNPs-NC nanocomposite are shown in Figure 2. Figure 2A shows the survey XPS spectra of the as-produced rGO/PdNPs-NC nanocomposite. The characteristic peaks evidenced the existence of elements such as C 1s, N 1s, Pd 3d, O 1s, and Pd 3p without highlighting the other detectable impurities, demonstrating the formation of the pure rGO/PdNPs-NC nanocomposite. Figure 2B shows the high-resolution C 1s XPS spectrum for the rGO/PdNPs-NC nanocomposite, in which four distinct electronic states are observed at 282.7, 283.3, 284.4, and 286.2 eV, conforming to the C–C, C–O, C=O, and O–C=O, respectively. The C 1s peaks associated with the O–C=O, C=O, and C–O groups decreased gradually as a result of the effective removal of most of the oxygen functional groups or species from the GO surface. These results further prove that GO has been converted into rGO during the formation of the rGO/PdNPs-NC nanocomposite in the presence of *Punica granatum* (pomegranate) peel extract as a green reducing and stabilizing agent. Figure 2C shows the XPS spectrum observed for the core level signal of O 1s, and it was further deconvoluted into two main peaks with their corresponding binding energy at 530.0 and 531.4 eV, which were assigned to C=O and C–OH/C–O–C, respectively.^66^

The Pd 3d core-level high-resolution XPS spectrum for the rGO/PdNPs-NC sample is shown in Figure 2D. The splitting patterns and binding energies of the Pd 3d signals for the rGO/PdNPs-NC sample can be resolved into doublet components 3d_5/2_ and 3d_3/2_ occurring due to the spin–orbital coupling of the Pd 3d atoms in response to the existence of Pd^0^ and Pd^2+^. The presence of the doublets at the relatively lower binding energy levels (335.9 and 341.5 eV) is ascribed to the metallic Pd, and the other doublet at 336.9 and 342.4 eV is assigned to the +2-oxidation state of Pd.^67,68^ It has been reported that noble metals such as Pd possess free mobility nature over the surfaces of reduced graphene oxide (rGO), which results in easy agglomeration of PdNPs. It is well known that Pd is an electron-deficient metal or material, which results in the transfer of π-electron or notable charge between the PdNPs and reduced graphene oxide (rGO) sheets, which represents that there are strong interactions between the PdNPs and supported reduced graphene oxide (rGO). Thus, the above-mentioned interactions successfully reduce the free mobility nature of the metal nanoparticles and help in maintaining the uniform distribution of nanoparticles on the surface of reduced graphene.^69,70^ Our results further support this argument.

#### 3.1.5 Scanning Electron Microscopy (SEM) and Energy-Dispersive X-ray Spectroscopy (EDS) Studies

**Figure 3:**
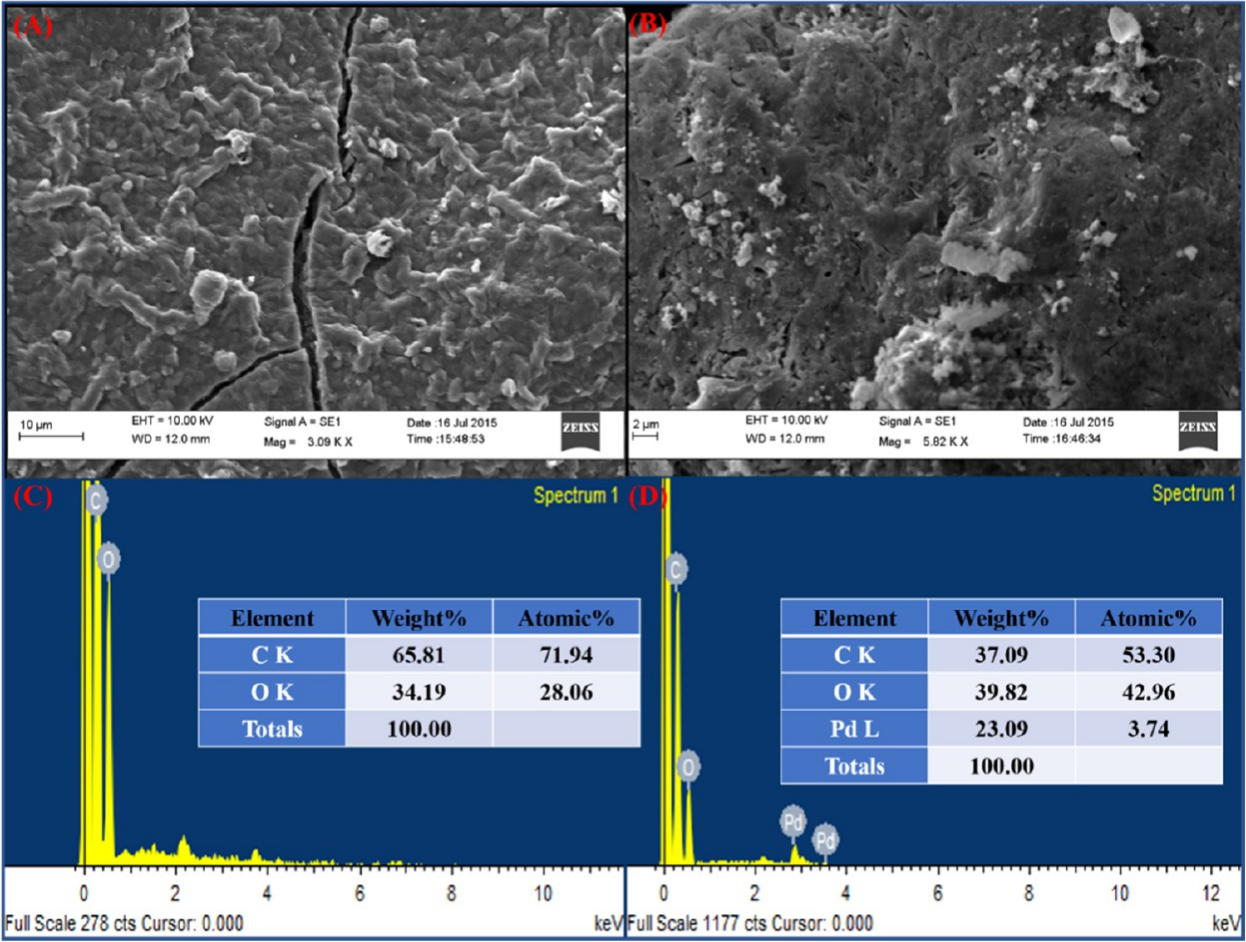
SEM images of rGO-PG (A) and rGO/PdNPs-NC (B). EDS spectra of rGO-PG (C) and rGO/PdNPs-NC (D).

The morphological features of the as-green synthesized rGO-PG and the rGO/PdNPs-NC using the *Punica granatum* (Pomegranate) peel extract were characterized by scanning electron microscopy (SEM). Figure 3A,B presents the representative SEM images of the green-reduced rGO-PG and rGO/PdNPs-NC, respectively. Figure 3A shows the typical SEM images of rGO-PG. The figure reveals that the surface of the reduced graphene oxide (rGO) nanosheets exhibits an aggregated structure, large surface area with wrinkled or crumpled texture-like morphology and entangled and rippled with each other, and also possess fully inflated curliness. These defects may have occurred due to the self-assembly of nanosheets via van der Waals forces during the deoxygenation process.^71^

Figure 3B displays the representative SEM micrograph for the rGO/PdNPs-NC, revealing fairly monodispersed PdNPs on the surface of the *Punica granatum* (pomegranate) peel extract-modified reduced graphene oxide (rGO). These results prove that PdNPs had strong affinity toward reduced graphene oxide (rGO). Energy-dispersive spectroscopy (EDS) analysis provides valuable information on the type of elemental composition present in the particular areas. The EDS spectra for the rGO-PG and rGO/PdNPs-NC samples are shown in Figure 3C,D. The detailed compositional data obtained from EDS spectra are illustrated as an inset table in the respective figures. The strong peak located at 3 keV evidences the existence of elemental PdNPs on the surface of reduced graphene oxide (rGO), which results in the formation of rGO/PdNPs-NC (Figure 3D). The most representative elements showed by the qualitative EDS spectral analysis were carbon and oxygen and Pd, and no other extra elements were traced out, indicating the high purity of the synthesized rGO-PG and rGO/PdNPs-NC samples. The weight percentages of “C”, “O”, and “Pd” determined from EDS spectra are listed in Table 1. According to it, the main composition of the rGO-PG sample is carbon (65.81%) and oxygen (34.19%) (see Figure 3C), whereas the main composition of the rGO/PdNPs-NC sample is carbon (37.09%), oxygen (39.82%), and Pd (23.09%) (see Figure 3D). These results reveal a good loading capacity of PdNPs on the surface of reduced graphene oxide (rGO) nanosheets of rGO/PdNPs-NC, and the obtained results are consistent with those of SEM observations.

**Table 1:** Elemental (“C”, “O”, and “Pd”) weight% and atomic% of the respective nanocomposites: rGO-PG and rGO/PdNPs-NC

sample	carbon (wt %)	carbon (atom %)	oxygen (wt %)	oxygen (atom %)	palladium (wt %)	palladium (atom %)
rGO-PG	65.81	71.94	34.19	28.06		
rGO/PdNPs-NC	37.09	53.30	39.82	42.96	23.09	03.74

#### 3.1.6 Transmission Electron Microscopy (TEM) and Selected-Area Electron Diffraction (SAED) Studies

**Figure 4:**
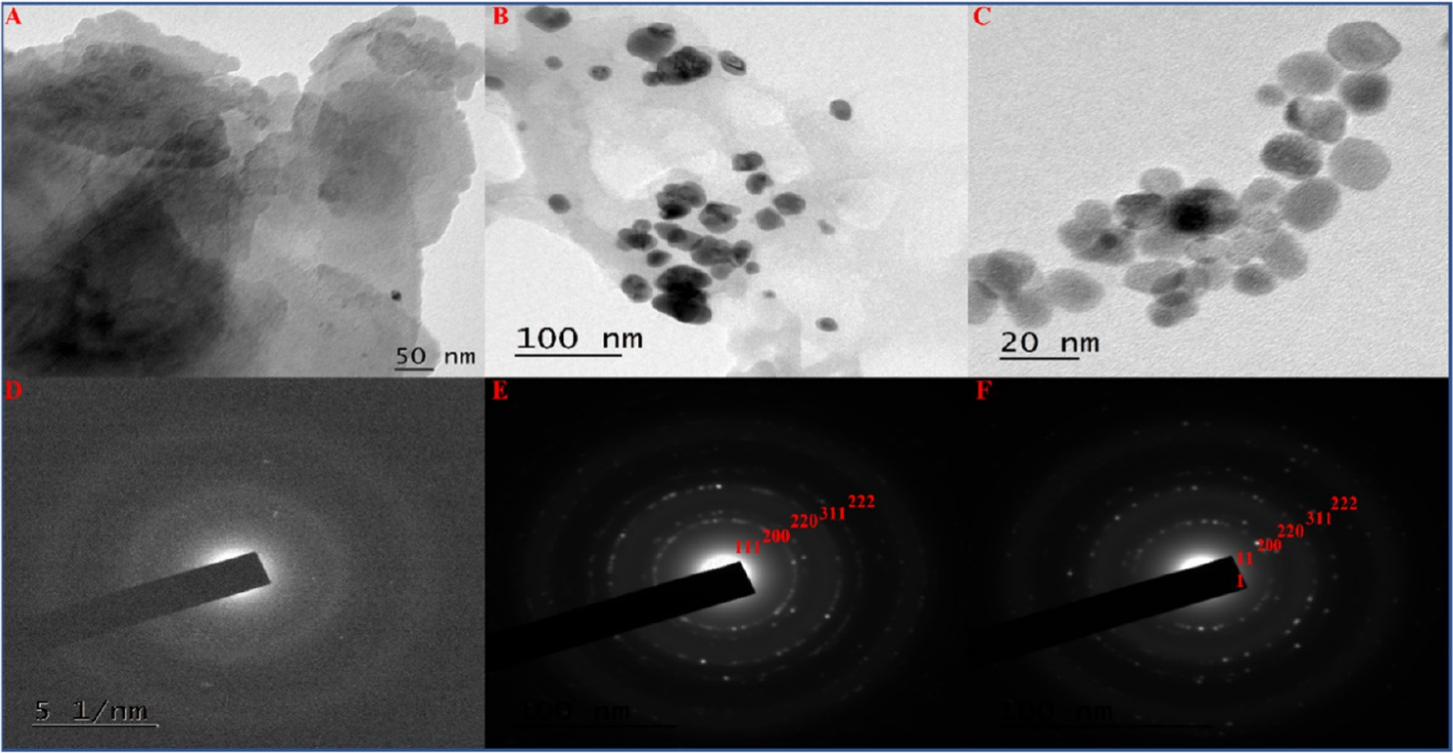
TEM images of rGO-PG (A), rGO/PdNPs-NC (B), and PdNPs. (C) SAED pattern of rGO-PG (D), rGO/PdNPs-NC (E), and PdNPs (F).

The detailed morphological properties such as structure, particle size, and crystallinity of rGO-PG, rGO/PdNPs-NC, and pure PdNPs were evaluated by transmission electron microscopy (TEM) analysis and selected-area electron diffraction (SAED) patterns for all the components (Figure 4). Figure 4A presents a typical TEM micrograph for the synthesized rGO-PG sample. It can be noted that the formed rGO-PG nanosheets exist in the form of well-exfoliated, translucent flat graphene layers with some corrugation on the rGO nanosheet surface, which resemble crumpled or wrinkled silk veil waves. This mainly occurs during the stabilization of the 2D membrane nanosheets, resulting in microscopic crumpling via buckling or bending movements of the sheets. This phenomenon is further supported by the fact that the complete exfoliation of coarse aggregates takes place on the surface of rGO.

Figure 4B shows the TEM image for the as-synthesized rGO/PdNPs-NC sample. As observed, the immobilized PdNPs are roughly spherical in shape with a mean particle size of 24 nm, and they are homogeneously dispersed on the wrinkled and crumpled solid surface of the as-synthesized rGO surface. The formation mechanism of the rGO/PdNPs-NC resulted in rGO with large surface area and uniformly distributed defective sites or active sites (known as dangling bonds) on rGO, which is a significant advantage for the effective anchoring of the PdNPs on the graphene surfaces. Moreover, the negatively charged oxygen functional moieties such as carboxyl, epoxy, carbonyl, and hydroxyl groups present on the GO sheets should act as nucleation sites for upcoming Pd^2+^ ions to produce PdNPs. It is interesting that Pd metal particles and graphene sheets do not appear as separate entities, but they are attached to reduced graphene oxide nanosheets. Hence, it is assumed that the Pd^2+^ ions firmly attach on the graphene nanosheets before undergoing the reduction process.^72^ These results also confirm that during the synthesis of rGO/PdNPs-NC involving the *Punica granatum* (Pomegranate) peel extract as a reducing agent, both graphene oxide (GO) and Pd ions undergo an *in situ* reduction process, resulting in Pd metal nanoparticles embedded on the surface of reduced graphene oxide layers.

It is worthwhile to note that the stabilization of the formed metal nanoparticles depends on the rehybridization of carbon in sp^2^ → sp^3^ in the graphene layer via metal–carbon bond formation (M–C), and the nucleation sites and homogenous dispersion of the metal clusters on the graphene are typically dominated by the presence of defective sites.^73−76^ Thus, the as-synthesized pure PdNPs were further characterized by TEM analysis in order to evaluate the morphology and size of the formed PdNPs. Figure 4C shows the TEM image of the pure PdNPs, which reveals almost uniform spherical shaped nanoparticles, with an average particle size of 6–19 nm (average ∼12 nm). Therefore, we can conclude that the green reducing agent *Punica granatum* (pomegranate) peel extract acts as a stabilizer and coater for the PdNPs.

On the other hand, the selected-area electron diffraction (SAED) patterns for the rGO-PG, rGO/PdNPs-NC, and pure PdNPs are shown in Figure 4D–F. Figure 4D shows the selected-area electron diffraction (SAED) pattern for the rGO-PG sample. It can be observed that the polycrystalline ring pattern is composed of many bright diffraction spots, which indicate the typical hexagonal symmetry of the newly formed carbon, typical of few layered graphene nanosheets. The selected-area diffraction analysis for the rGO/PdNPs-NC sample reveals resolved concentric rings with bright intermittent spots, confirming the high crystalline purity of the produced Pd nanoparticles. The diffraction patterns are broadly classified based on the PdNP crystallinity nature, and they match well with the JCPDS card No: 89–4897. The diffracted rings are ascribed to the crystallographic planes (111), (200), (220), (311), and (222) of the fcc PdNPs, and the obtained results agree well with the XRD result lattice plane of PdNPs. Hence, all these studies further confirm a good dispersion of PdNPs on the surface of the reduced graphene oxide (rGO) in the rGO/PdNPs-NC.

### 3.2 Evaluation of In Vivo Hepatoprotective Activity on Key Liver Function Parameters against Acetaminophen (APAP)-Induced Hepatotoxicity in Rats

**Figure 5:**
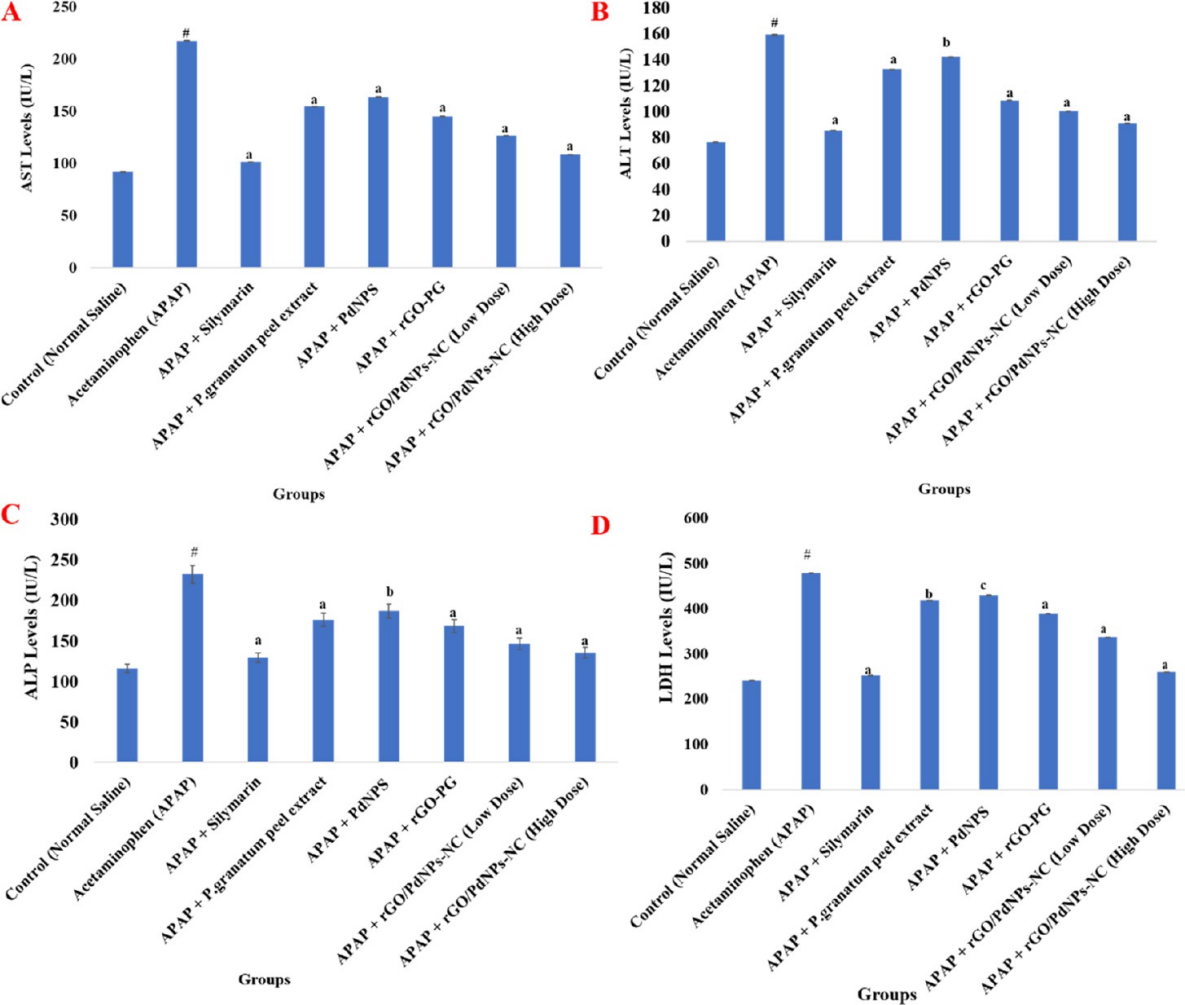
Effect of the *Punica granatum* (pomegranate) peel extract, PdNPs, rGO-PG, rGO/PdNPs-NC (low dose), rGO/PdNPs-NC (high dose), and positive control drug silymarin on the serum level of aspartate aminotransferase (AST) (A), alanine aminotransferase (ALT) (B), alkaline phosphatase (ALP) (C), and lactate dehydrogenase (LDH) (D) in acetaminophen-induced hepatic damage in rats, where control = normal saline (1 mL/kg b.w.), negative control = acetaminophen control (APAP) (2 g/kg b.w.), APAP + silymarin = (100 mg/kg b.w.), APAP + *Punica granatum* (Pomegranate) peel extract = (100 mg/kg b.w.), APAP + PdNPs = (100 mg/kg b.w.), APAP + rGO-PG = (50 mg/kg b.w.), APAP + rGO/PdNPs-NC (low dose) = (25 mg/kg b.w.), and APAP + rGO/PdNPs-NC (high dose) = (50 mg/kg b.w.). Values are expressed as the mean ± standard error of the mean (S.E.M) (*n* = 6) for each treatment; ^#^ Significant *P* < 0.001 for acetaminophen (group-II) compared with the normal control (group-I); ^a^ Significance *P* < 0.001, ^b^*P* < 0.01, and ^c^*P* < 0.05 for rest of the groups compared with the acetaminophen-treated group (group-II).

**Figure 6:**
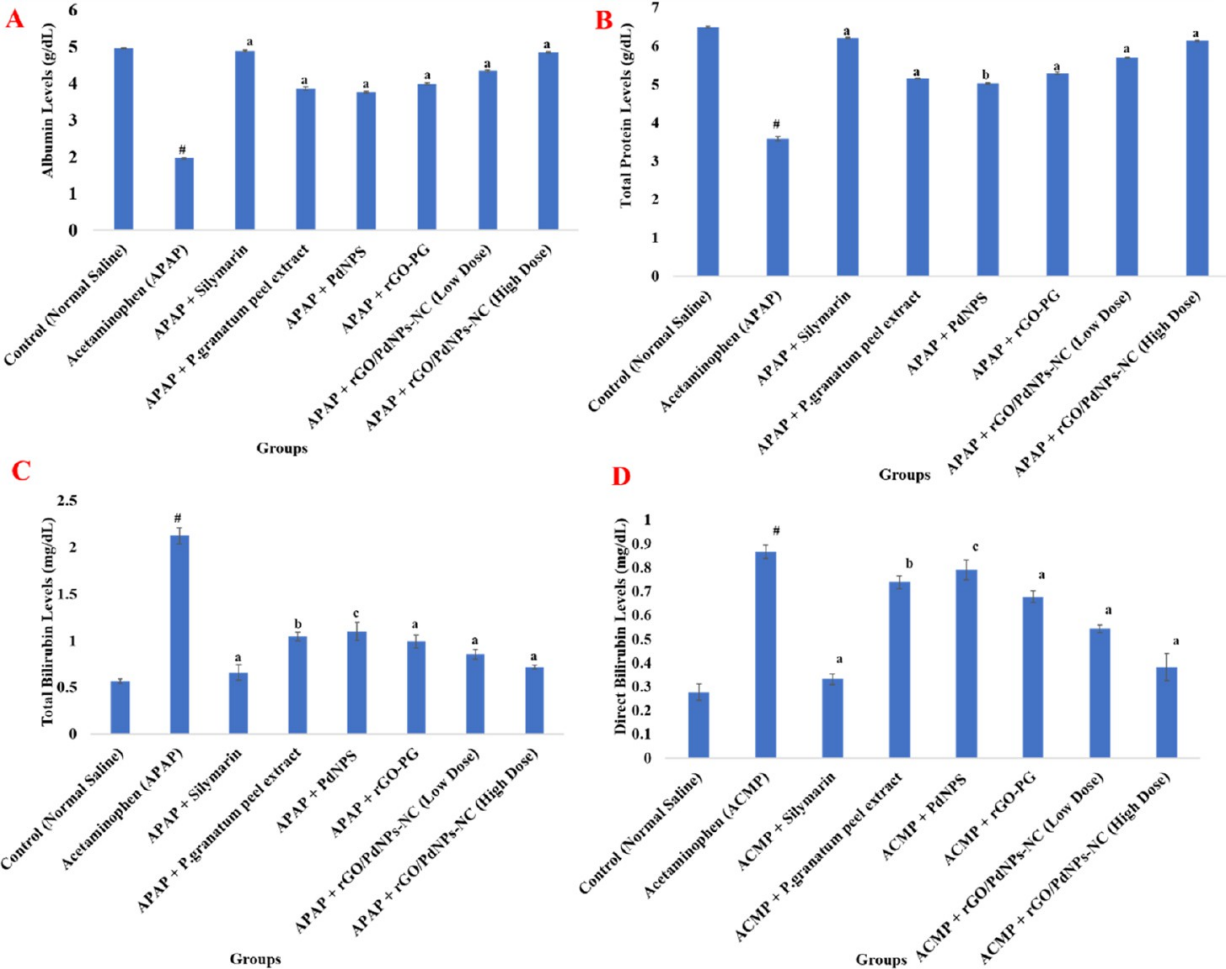
Effect of the *Punica granatum* (pomegranate) peel extract, PdNPs, rGO-PG, rGO/PdNPs-NC (low dose), rGO/PdNPs-NC (high dose), and positive control drug silymarin on the serum level of albumin (A), total protein (B), total bilirubin (C), and direct bilirubin (D) in acetaminophen-induced hepatic damage in rats, where control = normal saline (1 mL/kg b.w.), negative control = acetaminophen control (APAP) (2 g/kg b.w.), ACMP + silymarin = (100 mg/kg b.w.), APAP + *Punica granatum* (pomegranate) peel extract = (100 mg/kg b.w.), APAP + PdNPs = (100 mg/kg b.w.), APAP + rGO-PG = (50 mg/kg b.w.), APAP + rGO/PdNPs-NC (low dose) = (25 mg/kg b.w.), and APAP + rGO/PdNPs-NC (high dose) = (50 mg/kg b.w.). Values are expressed as the mean ± S.E.M. (*n* = 6) for each treatment; ^#^ Significant *P* < 0.001 for acetaminophen (group-II) compared with the normal control (group-I); ^a^ Significance *P* < 0.001, ^b^*P* < 0.01, and ^c^*P* < 0.05 for rest of the groups compared with the acetaminophen-treated group (group-II).

Therapeutic potentials of rGO/PdNPs-NC (low and high doses) also compared to those of lower potential abilities exhibited by some other components such as *Punica granatum* (pomegranate) peel extract, PdNPs, and rGO-PG along with the standard hepatoprotective potential drug silymarin were successfully evaluated through a short-term hepatoprotective study against acetaminophen-induced hepatotoxicity in the rat liver. In order to assess the degree of liver dysfunction in each group, various biochemical serum liver enzyme marker levels were recorded. A single overdose of (2 g/kg b.w.) acetaminophen through oral route administration significantly increased the serum levels of AST, ALT, ALP, LDH, total bilirubin, and direct bilirubin. Moreover, there is a decrease in serum levels of albumin and total proteins as observed in group-II acetaminophen (APAP), inducing toxicity in comparison to the normal control group-I (normal saline). These are listed in Tables 2, 3 and well illustrated in Figures 5 and 6. Results showed that hepatic injury induced by acetaminophen caused a significant rise in marker enzymes such as AST by 137.16%, ALT by 107.80%, ALP by 99.80%, LDH by 97.91%, total bilirubin (T.bil) by 271.93%, direct bilirubin (Di.bil) by 210.71%, albumin (ALB) by 60.20%, and total protein (T.P) by 44.84% compared to the normal control (group I). The percentage protection in marker enzymes of pretreated groups at their respective dosage concentrations such as *Punica granatum* (Pomegranate) peel extract (100 mg/kg b.w.), PdNPs (100 mg/kg b.w.), and rGO-PG (50 mg/kg b.w.) was as follows: AST 28.89% (*P* < 0.001), 24.80% (*P* < 0.001), 33.27% (*P* < 0.001), ALT 16.63% (*P* < 0.001), 10.65% (*P* < 0.01), 31.77% (*P* < 0.001), ALP 24.11% (*P* < 0.001), 19.40 (*P* < 0.01), 27.52% (*P* < 0.001), LDH 12.36% (*P* < 0.01), 9.93% (*P* < 0.05), 18.52% (*P* < 0.001), T.Bil 50.94% (*P* < 0.01) 48.11% (*P* < 0.05), 53.30% (*P* < 0.001), Di.Bil 14.94% (*P* < 0.01), 9.20% (*P* < 0.05), 21.83% (*P* < 0.001), ALB 95.94% (*P* < 0.001), 90.86% (*P* < 0.001), 102.54% (*P* < 0.001), and T.P 43.85% (*P* < 0.001), 40.22% (*P* < 0.01), 47.77% (*P* < 0.001) when compared to hepatotoxic control group-II acetaminophen (APAP). The maximum percentage protection in serum marker enzyme levels at the dose levels of rGO/PdNPs-NC (25 mg/kg b.w.) (low dose), rGO/PdNPs-NC (50 mg/kg b.w.) (high dose), and positive control drug silymarin (100 mg/kg b.w.) was as follows: AST 41.87% (*P* < 0.001), 50.20% (*P* < 0.001), 53.31% (*P* < 0.001), ALT 37.11% (*P* < 0.001), 42.91% (*P* < 0.001), 46.32% (*P* < 0.001), ALP 37.00% (*P* < 0.001), 41.56% (*P* < 0.001), 44.30% (*P* < 0.001), LDH 29.43% (*P* < 0.001), 45.60% (*P* < 0.001), 47.17% (*P* < 0.001), T.Bil 59.91% (*P* < 0.001), 66.04% (*P* < 0.001), 68.87% (*P* < 0.001), Di.Bil 37.93% (*P* < 0.001), 56.32% (*P* < 0.001), 62.07% (*P* < 0.001), ALB 120.30% (*P* < 0.001), 145.69% (*P* < 0.001), 147.72% (*P* < 0.001), and T.P 58.94% (*P* < 0.001), 71.51% (*P* < 0.001), 73.46% (*P* < 0.001). The above-obtained values are almost comparable to the treated group-III with silymarin, a standard potent hepatoprotective control drug (see Tables 2, 3 and Figures 5 and 6). Hence, the results indicate that pretreatment with both rGO/PdNPs-NC (25 mg/kg b.w., low dose) and (50 mg/kg b.w., high dose) significantly hinder leakage of intracellular hepatic enzymes into the serum (*P* < 0.001). Therefore, maintaining AST, ALT, ALP, LDH, total bilirubin, and direct bilirubin marker levels at lower ranges indicates the stability of biliary function. Moreover, the elevated serum protein levels suggest that stabilization of the endoplasmic reticulum takes place, which further supports increased generation of proteins that in turn enhance the hepatocyte regeneration capacity.

**Table 2:** Effect of the *Punica granatum* (Pomegranate) Peel Extract, PdNPs, rGO-PG, rGO/PdNPs-NC, and Silymarin on the Serum Level of AST, ALT, ALP, and LDH in Acetaminophen-Induced Hepatic Damage in Rats^a^

groups (*n* = 6)/design of treatment	AST (IU/L)	ALT (IU/L)	ALP (IU/L)	LDH (IU/L)
Group-I normal control (NC) (normal saline 1 mL/kg b.w.)	91.60 ± 0.18	76.49 ± 0.24	116.21 ± 5.43	241.37 ± 0.10
Group-II acetaminophen control (APAP) (2 g/kg b.w.)	217.24 ± 0.24^b^	158.95 ± 0.27^b^	232.19 ± 10.9^b^	477.69 ± 0.07^b^
Group-III APAP + silymarin (100 mg/kg b.w.)	101.42 ± 0.22^c^	85.32 ± 0.16^c^	129.32 ± 6.05^c^	252.38 ± 0.09^c^
APAP + *Punica granatum* (pomegranate) peel extract (100 mg/kg b.w.)	154.47 ± 0.20^c^	132.51 ± 0.18^c^	176.22 ± 8.23^c^	418.65 ± 0.19^d^
Group-V APAP + PdNPs (100 mg/kg b.w.)	163.37 ± 0.20^c^	142.02 ± 0.22^d^	187.14 ± 8.72^d^	430.25 ± 0.16^e^
Group-VI APAP + rGO-PG (50 mg/kg b.w.)	144.97 ± 0.21^c^	108.45 ± 0.24^c^	168.30 ± 7.86^c^	389.23 ± 0.16^c^
Group-VII APAP + rGO/PdNPs-NC (25 mg/kg b.w.)	126.28 ± 0.26^c^	99.97 ± 0.23^c^	146.26 ± 6.83^c^	337.11 ± 0.18^c^
Group-VIII APAP + rGO/PdNPs-NC (50 mg/kg b.w.)	108.19 ± 0.27^c^	90.75 ± 0.18^c^	135.68 ± 6.33^c^	259.76 ± 0.15^c^

a AST; aspartate aminotransferase, ALT; alanine aminotransferase, ALP; alkaline phosphatase, LDH; lactate dehydrogenase, and APAP; acetaminophen. Data represented in the table were expressed as the mean values ± standard error of the mean (S.E.M.). of six rats for each treatment.

b Significant *P* < 0.001 for acetaminophen (group-II) compared with the normal control (group-I).

c Significance *P* < 0.001.

d *P* < 0.01.

e *P* < 0.05 for rest of the groups compared with the acetaminophen-treated group (group-II).

**Table 3:** Effect of the *Punica granatum* (Pomegranate) Peel Extract, PdNPs, rGO-PG, rGO/PdNPs-NC, and Silymarin on the Serum level of Albumin, Total Protein, Total Bilirubin, and Direct Bilirubin in Acetaminophen-Induced Hepatic Damage in Rats^a^

groups (*n* = 6)/design of treatment	albumin (g/dL)	total protein (g/dL)	total bilirubin (mg/dL)	direct bilirubin (mg/dL)
Group-I normal control (NC) (normal saline 1 mL/kg b.w.)	4.95 ± 0.02	6.49 ± 0.02	0.57 ± 0.02	0.28 ± 0.03
Group-II Acetaminophen control (APAP) (2 g/kg b.w.)	1.97 ± 0.02^b^	3.58 ± 0.06^b^	2.12 ± 0.09^b^	0.87 ± 0.03^b^
Group-III APAP + Silymarin (100 mg/kg b.w.)	4.88 ± 0.02^c^	6.21 ± 0.01^c^	0.66 ± 0.08^c^	0.33 ± 0.02^c^
APAP + *Punica granatum* (Pomegranate) peel extract (100 mg/kg b.w.)	3.86 ± 0.04^c^	5.15 ± 0.02^c^	1.04 ± 0.05^d^	0.74 ± 0.03^d^
Group-V APAP + PdNPs (100 mg/kg b.w.)	3.76 ± 0.02^c^	5.02 ± 0.01^d^	1.10 ± 0.10^e^	0.79 ± 0.04^e^
Group-VI APAP + rGO-PG (50 mg/kg b.w.)	3.99 ± 0.02^c^	5.29 ± 0.02^c^	0.99 ± 0.07^c^	0.68 ± 0.03^c^
Group-VII APAP + rGO/PdNPs-NC (25 mg/kg b.w.)	4.34 ± 0.02^c^	5.69 ± 0.02^c^	0.85 ± 0.05^c^	0.54 ± 0.02^c^
Group-VIII APAP + rGO/PdNPs-NC (50 mg/kg b.w.)	4.84 ± 0.02^c^	6.14 ± 0.02^c^	0.72 ± 0.02^c^	0.38 ± 0.06^c^

a APAP; Acetaminophen. Data represented in the table were expressed as the mean values ± standard error of the mean (S.E.M.) of six rats for each treatment;.

b Significant *P* < 0.001 for acetaminophen (group-II) compared with the normal control (group-I);.

c Significance *P* < 0.001,.

d *P* < 0.01,.

e *P* < 0.05 for rest of the groups compared with the acetaminophen-treated group (group-II).

### 3.3 Evaluation of In Vivo Hepatoprotective Activity on Key Liver Antioxidant Parameters against Acetaminophen (APAP)-Induced Hepatotoxicity in Rats

**Figure 7:**
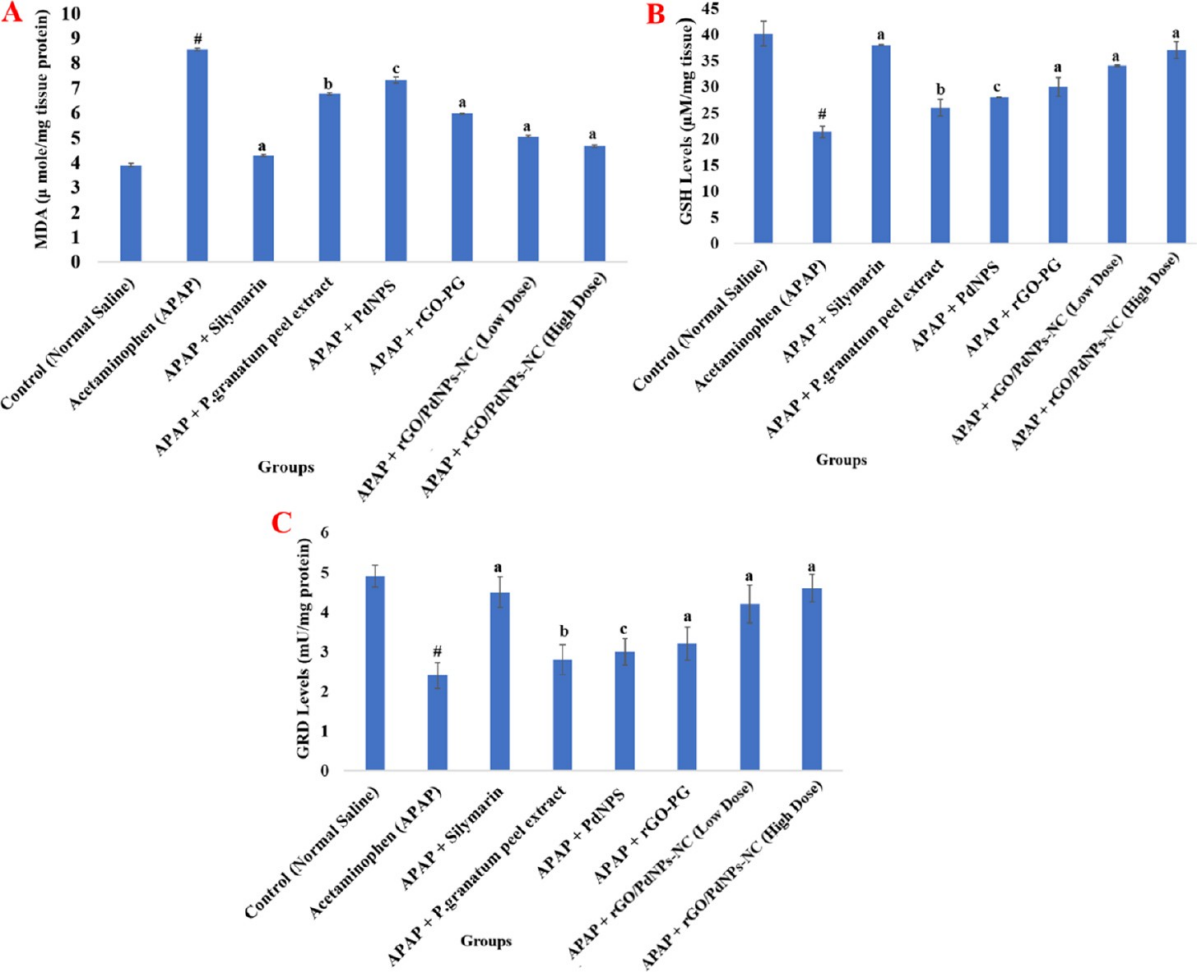
Effect of the *Punica granatum* (Pomegranate) peel extract, PdNPs, rGO-PG, rGO/PdNPs-NC (low dose), rGO/PdNPs-NC (high dose), and positive control drug silymarin on the liver tissue of APAP-intoxicated rats: malondialdehyde (MDA) (A), reduced glutathione (GSH) (B), and glutathione reductase (GRD) (C) where control = normal saline (1 mL/kg b.w.), negative control = acetaminophen control (APAP) (2 g/kg b.w.), ACMP + silymarin = (100 mg/kg b.w.), APAP + *Punica granatum* (pomegranate) peel extract = (100 mg/kg b.w.), APAP + PdNPs = (100 mg/kg b.w.), APAP + rGO-PG = (50 mg/kg b.w.), APAP + rGO/PdNPs-NC (low dose) = (25 mg/kg b.w.), and APAP + rGO/PdNPs-NC (high dose) = (50 mg/kg b.w.). Values are expressed as the mean ± S.E.M. (*n* = 6) for each treatment; ^#^ Significant *P* < 0.001 when acetaminophen (group-II) compared with the normal control (group-I); ^a^ Significance *P* < 0.001, ^b^*P* < 0.01, and ^c^*P* < 0.05 for the rest of the groups compared with the acetaminophen-treated group (group-II).

**Figure 8:**
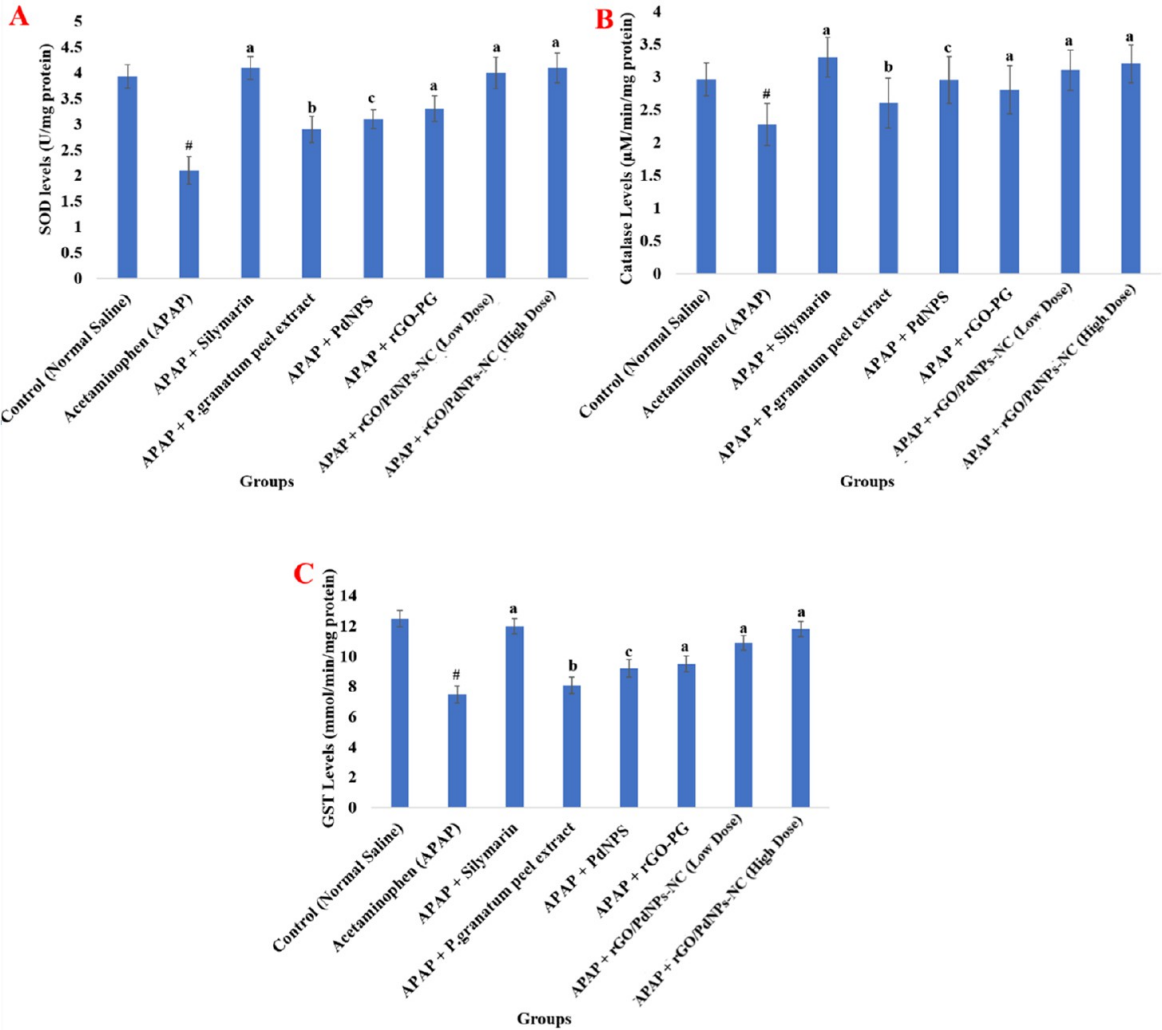
Effect of the *Punica granatum* (Pomegranate) peel extract, PdNPs, rGO-PG, rGO/PdNPs-NC (low dose), rGO/PdNPs-NC (high dose), and positive control drug silymarin on the liver tissue of APAP-intoxicated rats: superoxide dismutase (A), catalase (B), and glutathione-S-transferase (GST) (C) where: control = normal saline (1 mL/kg b.w.), negative control = acetaminophen control (APAP) (2 g/kg b.w.), ACMP + silymarin = (100 mg/kg b.w.), APAP + *Punica granatum* (pomegranate) peel extract = (100 mg/kg b.w.), APAP + PdNPs = (100 mg/kg b.w.), APAP + rGO-PG = (50 mg/kg b.w.), APAP + rGO/PdNPs-NC (low dose) = (25 mg/kg b.w.), and APAP + rGO/PdNPs-NC (high dose) = (50 mg/kg b.w.). Values are expressed as the mean ± S.E.M. (*n* = 6) for each treatment; ^#^ Significant *P* < 0.001 when acetaminophen (group-II) compared with the normal control (group-I); ^a^ Significance *P* < 0.001, ^b^*P* < 0.01, and ^c^*P* < 0.05 for the rest of the groups compared with the acetaminophen-treated group (group-II).

A single oral acute toxicity application of acetaminophen in Wistar albino rats also evidenced hepatotoxicity in antioxidant parameters of rat liver tissue, as indicated by the significant decrease in GSH, GRD, SOD, CAT, and GST from 40.16 ± 2.33 (μM/mg tissue), 4.9 ± 0.27 (mU/mg protein), 3.93 ± 0.23 (U/mg protein), 2.96 ± 0.25 (μM/min/mg protein), and 12.5 ± 0.54 (mmol/min/mg protein) in the normal control group to 21.39 ± 1.10 (μM/mg tissue), 2.4 ± 0.32 (mU/mg protein), 2.1 ± 0.27 (U/mg protein), 2.27 ± 0.32 (μM/min/mg protein), and 7.5 ± 0.56 (mmol/min/mg protein) in the acetaminophen-treated group and the noticeable increase in the content of oxidative marker of malondialdehyde (MDA) from 3.894 ± 0.0519 (μ mole/mg tissue protein) to 8.5476 ± 0.0402 (*p* < 0.001, see Figures 7 and 8). Administration of some other components such as *Punica granatum* (pomegranate) peel extract, PdNPs, rGO-PG, and standard hepatoprotective potential drug silymarin and along with the hepatoprotective testing components rGO/PdNPs-NC (25 mg/kg b.w., low dose) and rGO/PdNPs-NC (50 mg/kg b.w. high dose) once daily for 8 consecutive days prior to the single overdose of (2 g/kg b.w.) acetaminophen through oral route administration effectively protected against a decrease in hepatic GSH, GRD, SOD, CAT, and GST, considered as an index of the antioxidant status of tissues. As represented in Figures 7 and 8, there is a significant increase in these antioxidant enzyme activities and there is also an observable sharp decrease in the malondialdehyde (MDA) level in the Wistar rats treated with rGO/PdNPs-NC (25 mg/kg b.w., low dose) and rGO/PdNPs-NC (50 mg/kg b.w., high dose) (*p* < 0.001) relative to the acetaminophen (group-II) single treatment.

### 3.4 Histopathological Examination of the Liver Tissue Section against Acetaminophen (APAP)-Intoxicated Liver Injury

**Figure 9:**
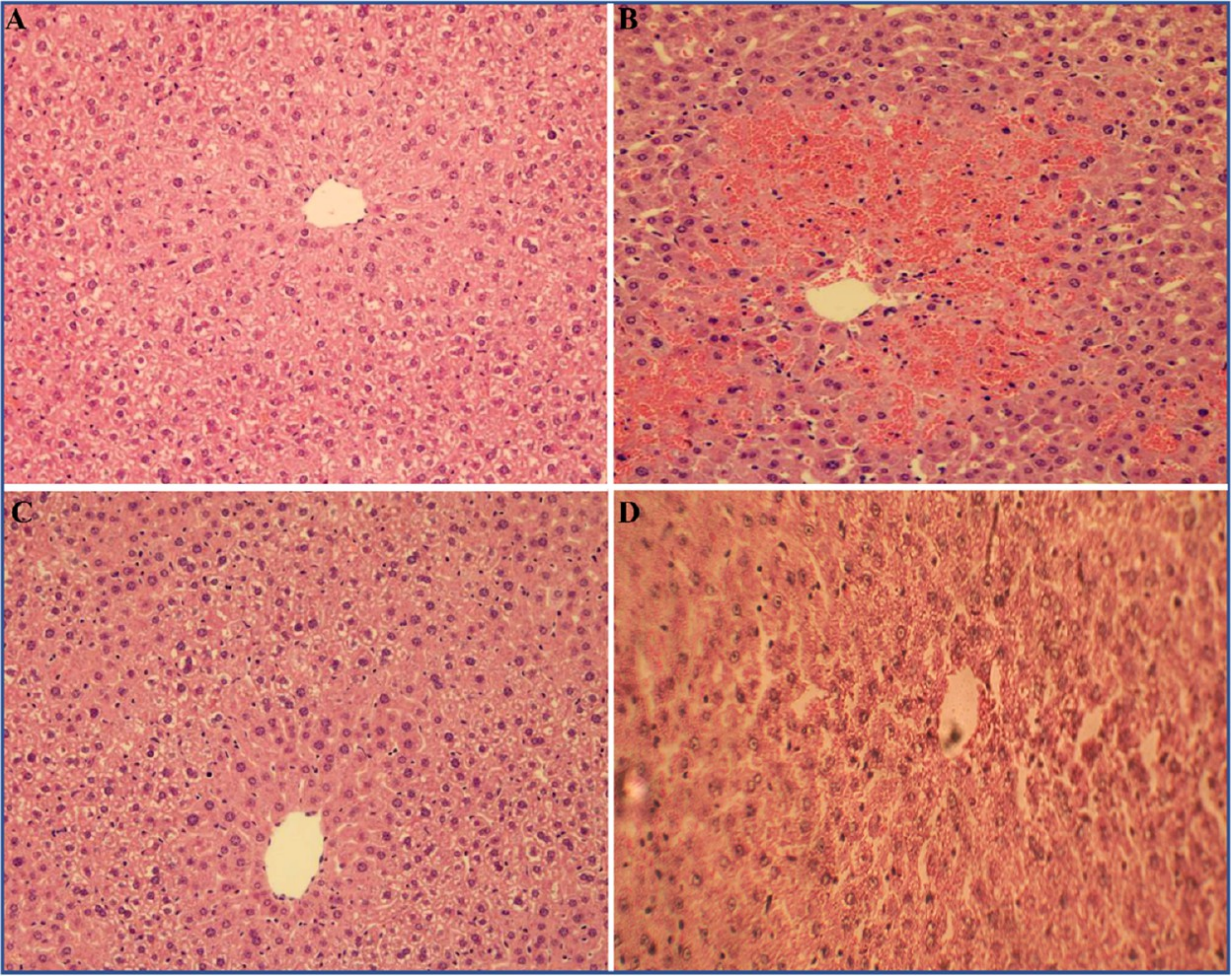
Histopathological examination of photomicrographs of the rat’s liver tissue sections belonging to different groups stained with hematoxylin and eosin: liver section of normal control rats treated with normal saline (1 mL/kg b.w.) (A), liver section of rats treated with acetaminophen (APAP) (2 g/kg b.w.) alone (B), liver section of rats treated with the positive control drug silymarin (100 mg/kg b.w.) + acetaminophen (APAP) (C); and liver section of rats treated with the aqueous *Punica granatum* (pomegranate) peel extract (100 mg/kg b.w.) (D).

**Figure 10:**
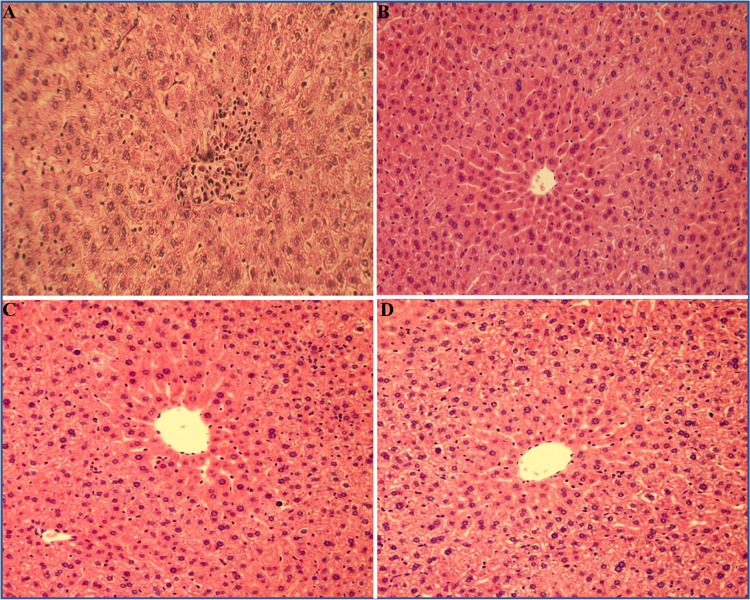
Histopathological examination of photomicrographs of the rat’s liver tissue sections belonging to different groups stained with hematoxylin and eosin: liver section of rats treated with PdNPs (100 mg/kg b.w.) + acetaminophen (APAP) (A); liver section of rats treated with rGO-PG (50 mg/kg b.w.) + acetaminophen (APAP) (B); liver section of rats treated with rGO/PdNPs-NC (25 mg/kg b.w.) (low dose) + acetaminophen (APAP) (C); and liver section of rats treated with rGO/PdNPs-NC (50 mg/kg b.w.) (high dose) + acetaminophen (APAP) (D).

The results of histopathological examination data collected further for different groups of rats treated with various components such as normal saline, GO, rGO-PG, *Punica granatum* (pomegranate) peel extract, PdNPs, rGO/PdNPs-NC, acetaminophen (APAP), and positive control drug silymarin and their respective liver tissue section basically provided supportive evidence for the biochemical serum enzyme assay analysis, as represented in Figures 9 and 10. Histopathological observation of the liver section in normal control rats exhibited well-organized hepatic lobules, which are roughly polygonal/hexagonal in shape, within each lobule. Hepatic cells are systematically arranged into hepatic cords, which run radiantly around the central vein, hepatocytes with well-preserved acidophilic cytoplasm and centrally positioned, regular-sized prominent nuclei and normal phagocytic Kupffer cells. Moreover, there is no evidence of cell replacement or regeneration changes, inflammation, fibrosis, necrosis, or toxic changes as observed in Figure 9A. In contrast, the acetaminophen (APAP)-intoxicated rats showed liver sections with severe damage that leads to complete loss of degeneration and disarrangement of the normal hepatic cellular architecture with massive intrahepatic hemorrhagic necrosis around the centrilobular region (see Figure 9B). The hepatocytes with vacuolization and swollen cytoplasm were observed, while the nuclei were deeply stained and shrunken, and broad inflammatory cell infiltration of lymphocytes and diffused hyperplasia Kupffer cells around the central vein and finally the loss of cellular boundaries are noticed in Figure 9B. However, the liver section of rats administered with the positive control drug silymarin at the dose levels of 100 mg/kg b.w. and intoxicated with acetaminophen (APAP) depicts almost normal hepatic cellular architecture, with very minute or lesser degeneration and disarrangement of hepatocytes, representing a marked level of regeneration activity (see Figure 9C). Histology of the liver section of rats treated with aqueous *Punica granatum* (pomegranate) peel extract (100 mg/kg b.w.) and intoxicated with acetaminophen (APAP) showed moderate hepatoprotective activity with a very mild degree of fatty changes and necrosis, and a small portion of hepatocytes in the centrilobular region appear to be slightly swollen, and vacuolization of the cytoplasm takes place (see Figure 9D). In contrast, the liver section of the rats treated with PdNPs (100 mg/kg b.w.) and intoxicated with acetaminophen (APAP) showed the absence of cellular necrosis but with minimal inflammatory and hepatic cellular infiltration in liver tissues around the central vein region (see Figure 10A). On the other hand, liver sections of the rats treated with rGO-PG (50 mg/kg b.w.) and intoxicated with acetaminophen (APAP) exhibited a potential hepatoprotective activity with very minute degenerative changes like slight swelling of hepatic cells in the centrilobular region, and the hepatocyte architecture appears to be like that of normal hepatocytes (see Figure 10B), whereas the rGO/PdNPs-NC (low dose) (25 mg/kg b.w.) and rGO/PdNPs-NC (high dose, 50 mg/kg b.w.) and intoxication with acetaminophen (APAP) showed the ability of regeneration of hepatic cells around the central vein almost equal to that of the normal hepatocellular architecture, indicating higher hepatoprotective action. Interestingly, the administration of rGO/PdNPs-NC at a dose of 50 mg/kg b.w. was more effective in terms of the highest recovery potential of hepatic cellular architectures with no signs of cellular necrosis, fibrosis, inflammation, and infiltration of lymphocytes and hepatocyte degeneration and disarrangement, when compared to that of rGO/PdNPs-NC at a dose level of 25 mg/kg b.w. (see Figure 10C,D). Overall, the histopathological observations corroborate well with the biochemical serum enzyme assay analysis and further confirm that rGO/PdNPs-NC has the tendency to reduce the degree of acetaminophen-intoxicated liver injury.

## 4 Discussion

The current study mainly focuses on the green-synthesized rGO/PdNPs-NC, the produced nanocomposite (rGO/PdNPs-NC) at 25 mg/kg b.w. (low dose) and 50 mg/kg b.w. (high dose) along with other relative components and their respective dosage concentration levels such as rGO-PG (50 mg/kg b.w.), *Punica granatum* (pomegranate) peel extract (100 mg/kg b.w.), PdNPs (100 mg/kg b.w.), and positive control drug silymarin (100 mg/kg b.w.). They were thoroughly investigated and evaluated further against acetaminophen (APAP)-induced hepatotoxicity (2 g/kg b.w.) in Wistar albino rats.

A three-month study of palladium exposure (10–250 μg/L) through drinking water in male rats reported that palladium accumulated mainly in the kidney, but not in the liver, lung, spleen, or bones, while elimination occurred through the fecal route.^77^ Another study found that palladium(II) chloride (PdCl_2_) was poorly absorbed (<0.5%) from the digestive tract, i.e., 95% of the palladium was eliminated in the feces of rats due to nonabsorption.^78^ Hence, oral administration of paracetamol (2 g/kg b.w.) on the 8th day in a single dose^79,80^ or 3 g/kg b.w. on the 8th day in a single dose^7,81,82^ was given, and after 24 h of paracetamol administration orally, the blood was obtained through the retro-orbital plexus under light anesthesia, and the animals were sacrificed. The obtained results provide a piece of scientific evidence and also demonstrate the effective hepatoprotective role played by rGO/PdNPs-NC when compared to other components.

The rGO/PdNPs-NC was synthesized using the *Punica granatum* (pomegranate) peel extract, which acted as both a reducing and capping reagent. This greener synthesis approach method involves many other added advantages such as rapid production of products with a lower cost effect and environmental friendliness and can be easily scaled up for larger scale synthesis within lesser time. In order to achieve our goal, the green-synthesized rGO/PdNPs-NC was subjected to many physical and chemical characterizations by using different analytical techniques such as XRD, FTIR, Raman spectroscopy, XPS spectroscopy, SEM, EDS, TEM, and SAED pattern analysis. The XRD analysis reveals that there is a notable decrease in the peak intensity, indicating the successful reduction of GO and conversion into fruitful rGO-PG by utilizing the *Punica granatum* (pomegranate) peel extract, determining that the oxygen-containing moieties present on the GO surface were effectively reduced. In the case of rGO/PdNPs-NC, the fcc phase of PdNP nanocrystals was decorated on the rGO surfaces. The FTIR spectroscopy technique depicts that the oxygen-containing functional groups such as carboxyl, epoxy, and hydroxyl groups were abundantly found on the GO surface. After conversion of GO to rGO-PG, these oxygen moieties were successfully eliminated via a biological reduction process. The Raman spectroscopy technique suggests that defects persist on the carbon surfaces, and these defective sites help in anchoring of nucleated PdNPs on to the surface of reduced graphene oxide (rGO) nanosheets. Moreover, re-establishment of the numerous conjugated graphene networks, especially sp^2^ carbon, was observed, which denotes that the rGO/PdNPs-NC was well established, where pure fcc PdNPs are dispersed on graphene nanosheets. X-ray photoelectron spectroscopy (XPS) deals with the type of elements and their respective elemental chemical oxidation states that got incorporated or distributed onto the surface of reduced graphene oxide nanosheets. The rGO/PdNPs-NC presents elements such as carbon, oxygen, and palladium that exists in both Pd^2+^ and Pd^0^ oxidation states. SEM and TEM results denote the morphology, particle size, and crystallinity of the formed nanocomposites and pure PdNPs. The above-mentioned results suggest that reduced graphene oxide sheets exhibit wrinkled, crumpled, and corrugated morphology, while in rGO/PdNPs-NC, the uniform distribution of PdNPs with a roughly spherical shape and a particle size of 24 nm is observed on the surface of rGO nanosheets. The pure PdNPs also possess a spherical morphology with an average particle size of 6–19 nm. EDS data reveal that the synthesized GO, rGO-PG, and rGO/PdNPs-NC are composed of “C”, “O”, and Pd as main elements without other elements, which indicates the high purity of the synthesized nanomaterials. The SAED pattern reveals that the formed rGO-PG, rGO/PdNPs-NC, and pure PdNPs are polycrystalline in nature. In the case of pure PdNPs and rGO/PdNPs-NC, the resolved diffracted rings originating are ascribed to the crystallographic planes (111), (200), (220), (311), and (222) of the fcc PdNPs. The above-mentioned results demonstrate that the PdNPs are uniformly decorated on the rGO nanosheet surface, which ultimately leads to the successful synthesis of rGO/PdNPs-NC.

The liver is considered the major vital organ of an organism; its prime responsibility is regulating the body homeostasis, besides it also plays a fundamental role in inflammatory, detoxification, excretion, and metabolism responses.^83^ The metabolism of carbohydrates, lipids, serum proteins, and bilirubin occurs in the liver cells of the reticuloendothelial system. Therefore, it is a well known primary site for metabolism. Hence, this metabolic process is coined as hepatic metabolism.^84−86^ Other extrahepatic metabolism sites are readily available, which include the organs including the kidney, lungs, gastrointestinal tract epithelial cells, skin, placenta, and adrenals. However, the cytochrome distribution content in liver cells/tissues is found to be higher when compared to other organs, such as kidney, intestine, and lungs.^87^ The liver is such a versatile organ present in the body mainly concerned with detoxification and excretion of many exogenous and endogenous compounds, xenobiotics, antibiotics, other toxic chemicals, etc., and it also regularizes the internal chemical environment. Hence, the hepatic system was designed in such a manner where it not only performs its own physiological functions but also develops and orchestrates a superior self-defensive/protective mechanism within them. Therefore, any unwanted damage caused to the liver, especially by hepatotoxic reagents, leads to a grave consequence or shows impact on human health conditions.

Drug-induced hepatotoxicity is one of the major open challenges faced by most of the physicians in today’s world because it is mainly caused due to the frequent usage of medications associated with drug molecules/moieties, antibiotics, antitubercular chemotherapeutics, and nonsteroidal anti-inflammatory drugs. These drugs have the capacity/ability to induce hepatotoxicity in idiosyncratic mode. These above-mentioned changes ultimately result in the development of acute liver failure cases, and sometimes, they even cause adverse effects such as morbidity and mortality. Acetaminophen has been provided with a warning label, which indicates that there is a possibility to induce serious hepatotoxicity in exceeding doses.^88^ Acetaminophen (APAP)-induced experimental hepatotoxicity has served as a mechanically well-studied and most commonly used reliable model for screening the efficacy or therapeutic potential of synthetic drugs. As per literature data, many similarities exist between human and rodent physiologies; the rodent animal model acted as a suitable source for better understanding the etiology and pathogenesis of hepatotoxicity.^89,90^

Acetaminophen (paracetamol) is a widely known antipyretic and analgesic drug that can be safe at therapeutic doses but can cause hepatic damage in both humans and animals and can be fatal sometimes at higher toxic doses. The mechanism or bioactivation of acetaminophen-induced hepatocellular injury involves the conversion of the highly reactive and cytotoxic intermediate metabolite *N*-acetyl-*para*-benoquinonimine (NAPQI). Usually, acetaminophen is metabolized primarily via cytochrome P-450 to generate the highly reactive electrophilic NAPQI.^91^ The formed electrophile is eliminated by conjugation with glutathione (GSH) and metabolized to a mercapturic acid, which is in a safer form and excreted in the urine.^18^ Therefore, in the present study, acetaminophen was employed as an induced toxic agent in Wistar rats, and the hepatoprotective activity of rGO/PdNPs-NC at 25 mg/kg b.w. (low dose) and 50 mg/kg b.w. (high dose) and other relative compounds such as *Punica granatum* (pomegranate) peel extract (100 mg/kg b.w.), PdNPs (100 mg/kg b.w.) rGO-PG (50 mg/kg b.w.), and the positive control drug silymarin (100 mg/kg b.w.) was thoroughly studied. Since the synthesized rGO/PdNPs-NC by *Punica granatum* peel extracts is small in size (less than 100 nm), it could be eliminated through the liver, either by liver hepatocytes via phagocytic Kupffer cells or the biliary system, whereas other smaller size nanocomposites are likely excreted in urine, which enables their faster elimination from the body, thereby preventing excessive accumulation in body tissues and organs. The extent of toxicity was estimated by biochemical enzyme markers such as SGOT, SGPT, ALP, LDH, ALB, total protein, total bilirubin, and direct bilirubin. The *Punica granatum* (pomegranate) peel extracts possess many phytochemicals, i.e., secondary metabolites such as different polyphenols (cyanidin, delphinidin, ellagic acid, pelargonidin, hydrolyzable tannins, 3,5-diglucosides, and 3-glucosides), antioxidants (punicalin, punicalagin, and gallagic acid), and other polyhydroxy-functional moieties such as free ellagic acid, ellagic acid glycosides, ellagitannins, gallotannins, punicalagin, and punicalagin. These oxidized polyphenols are uniformly distributed onto the surface of rGO-PG via van der Waals (π–π stacking) interactions. Moreover, the PdNPs doped on the rough surface of the rGO-PG also exhibit a high surface-to-volume ratio and surface plasma resonance (SPR) as well as synergistic effects. Therefore, the formed rGO/PdNPs-NC product has higher potency toward free radical scavenging activity, which results in the systematic controlling of the serum enzymatic levels with hepatoprotective activity toward hepatocytes.

The results of the present study clearly demonstrate that the serum levels of the hepatic enzymes SGOT, SGPT, ALT, LDH, and bilirubin were drastically increased and those of ALB and TP decrease, reflecting that the hepatocellular damage occurred in the acetaminophen-induced hepatotoxicity animal model. Furthermore, an obvious sign of hepatic injury is proved by the appearance of these hepatic biochemical enzymes in the blood stream, as they initially occur in the cytoplasm. Moreover, bilirubin is a byproduct of heme within the reticuloendothelial system; its elevation levels in the blood stream can lead to overproduction, increased hemolysis, which in turn decreases conjugation or impaired bilirubin transportation.^92^ Bilirubin is used as an index to assess the normal functioning of the liver and to identify the extent of hepatocellular injury.

Antioxidant enzymes such as catalase (CAT), superoxide dismutase (SOD), reduced glutathione (GSH), glutathione reductase (GRD), and glutathione-S-transferase (GST) are very important in protecting organisms from reactive oxygen species (ROS). Catalase is a hemeprotein found predominantly in peroxisomes of eukaryotic cells that actively catalyzes the conversion of hydrogen peroxide to oxygen and water. Superoxide dismutase (SOD) is a defense enzyme, which converts superoxide radicals to hydrogen peroxide. Glutathione is the most abundant naturally existing tripeptide; nonenzyme biological antioxidants are present in the liver. Its major function is removing free radicals such as superoxide radicals and hydrogen peroxide. It is also involved in the maintenance of membrane protein detoxification of xenobiotics and biotransformation of drugs.^93^ Malondialdehyde (MDA) is a major reactive aldehyde that rises during the final stages of lipid peroxidation of the polyunsaturated fatty acid of the biological membrane. Interestingly, it is to be elucidated that acetaminophen-induced hepatotoxicity significantly reduces SOD, CAT, GSH, GRD, and GST activities, which implies severe damage to the liver caused by acetaminophen. The induction of acetaminophen results in the generation of lipid peroxidation induced by the free radicals derived from acetaminophen. Therefore, the antioxidant activity must be more potent to hinder the free radical production to prevent acetaminophen hepatopathies. There is also a significant decrease in GSH levels, while it is depleted in the acetaminophen-treated group because the conjugation of glutathione with NAPQI might form a safer component known as mercapturic acid.

*N*-Acetyl-*p*-benzoquinoneimine depletes GSH through covalent bonding with sulfhydryl groups and interacts with cell proteins, causing damage to the transport system as well as the membrane of hepatocytes with leakage of different cytosolic enzymes into the blood and enhanced enzyme levels in the serum.^7,82,94^ Also, NAPQI produces ROS, which cause oxidative damage of proteins, forming protein carbonyls (PCOs). This could be recovered by regeneration of hepatocytes through the synthesis of proteins needed for normalization or elimination of toxic metabolites or their detoxification. Under this circumstance, the rGO/PdNPs-NC may form a complex with NAPQI through carbonyl groups to electron-deficient palladium and deactivates its toxicity. As a result, the injured hepatocytes are recovered by improving the synthesis of proteins, reducing the ROS generation and repairing of the hepatocyte membrane. Further studies are going on to find out the actual bonding and its mechanism of action in alleviating liver injury and related ailments.

Histopathological examination also provides another piece of visual evidence for the hepatoprotective effects of the targeted investigated components such as rGO/PdNPs-NC 25 mg/kg b.w. (low dose) and 50 mg/kg b.w. (high dose) and other relative compounds such as *Punica granatum* (pomegranate) peel extract (100 mg/kg b.w.), PdNPs (100 mg/kg b.w.), rGO-PG (50 mg/kg b.w.), and positive control drug silymarin (100 mg/kg b.w.). These results are in agreement with the biochemical serum enzyme parameter assay and liver tissue; the histological alteration is induced by acetaminophen. The changes are severe ballooning, cytolysis, pyknosis, infiltration, and inflammatory cells as previously reported.^95−97^

## 5 Conclusions

In summary, the effect of the green-synthesized rGO/PdNPs-NC produced using the *Punica granatum* (pomegranate) peel extract as a suitable reducing and stabilizing agent was evaluated on acetaminophen-induced hepatotoxicity in Wistar albino rats. The results showed that rGO/PdNPs-NC 50 mg/kg b.w. (high dose) exhibited a protective effect against acetaminophen-induced hepatotoxicity in Wistar rats depicted by the significant decrease in AST, ALT, LDH, ALP, and bilirubin. ALB, total protein concentration, and liver antioxidant enzymes such as SOD, CAT, GSH, GRD, and GST were significantly elevated, while a decrease in MDA levels was observed. In addition, the control of acetaminophen-induced histopathological changes in the liver was observed. Further investigation is needed to identify the exact phytoconstituents responsible for reduction and stabilization of the synthesized nanocomposites as well as the mechanism of their hepatoprotective effect.

## Notes

XRD patterns, FTIR spectra, and Raman spectra of graphene oxide (GO), reduced graphene oxide (rGO-PG), PdNPs, and reduced graphene oxide palladium nanoparticle nanocomposite (rGO/PdNPs-NC) to characterize them; XPS and overall XPS survey spectra of rGO/PdNPs-NC to know the oxidation state of the elements; SEM images and EDS spectra of rGO-PG and rGO/PdNPs-NC to know the morphology and elementary composition; TEM images and SAED patterns of rGO-PG, rGO/PdNPs-NC, and PdNPs to know the size and crystallinity; effect of the *Punica granatum* (pomegranate) peel extract, PdNPs, rGO-PG, rGO/PdNPs-NC (low dose), rGO/PdNPs-NC (high dose), and positive control drug silymarin on the serum level of aspartate aminotransferase (AST), alanine aminotransferase (ALT), alkaline phosphatase (ALP), and lactate dehydrogenase (LDH) in acetaminophen-induced hepatic damage in rats; effect of the *Punica granatum* (pomegranate) peel extract, PdNPs, rGO-PG, rGO/PdNPs-NC (low dose), rGO/PdNPs-NC (high dose), and positive control drug silymarin on the serum level of albumin, total protein, total bilirubin, and direct bilirubin in acetaminophen-induced hepatic damage in rats; effect of the *Punica granatum* (pomegranate) peel extract, PdNPs, rGO-PG, rGO/PdNPs-NC (low dose), rGO/PdNPs-NC (high dose), and positive control drug silymarin on the liver tissue of APAP-intoxicated rat malondialdehyde (MDA), reduced glutathione (GSH), and glutathione reductase (GRD); effect of the *Punica granatum* (pomegranate) peel extract, PdNPs, rGO-PG, rGO/PdNPs-NC (low dose), rGO/PdNPs-NC (high dose), and positive control drug silymarin on the liver tissue of APAP-intoxicated rat superoxide dismutase, catalase, and glutathione-*S*-transferase (GST); histopathological examination of photomicrographs of the rat’s liver tissue sections belonging to different groups stained with hematoxylin–eosin: liver section of normal control rats treated with (normal saline 1 mL/kg b.w.), acetaminophen (APAP) (2 g/kg b.w.) alone, positive control drug silymarin (100 mg/kg b.w.) + acetaminophen (APAP), and aqueous *Punica granatum* (pomegranate) peel extract (100 mg/kg b.w.); and histopathological examination of photomicrographs of the rat’s liver tissue sections belonging to different groups stained with hematoxylin–eosin: liver section of rats treated with PdNPs (100 mg/kg b.w.) + acetaminophen (APAP), rGO-PG (50 mg/kg b.w.) + acetaminophen (APAP), rGO/PdNPs-NC (25 mg/kg b.w.) (low dose) + acetaminophen (APAP), and rGO/PdNPs-NC (50 mg/kg b.w.) (high dose) + acetaminophen (APAP).
